# Supplementary material for: The baseless mutant links protein phosphatase 2A with basal cell identity in the brown alga Ectocarpus
Source: Development. 2023 Feb 14;150(4):dev201283. doi: 10.1242/dev.201283 (PMC10112911; doi:10.1242/dev.201283)
Supplement: Supplementary information [file develop-150-201283-s1.pdf]

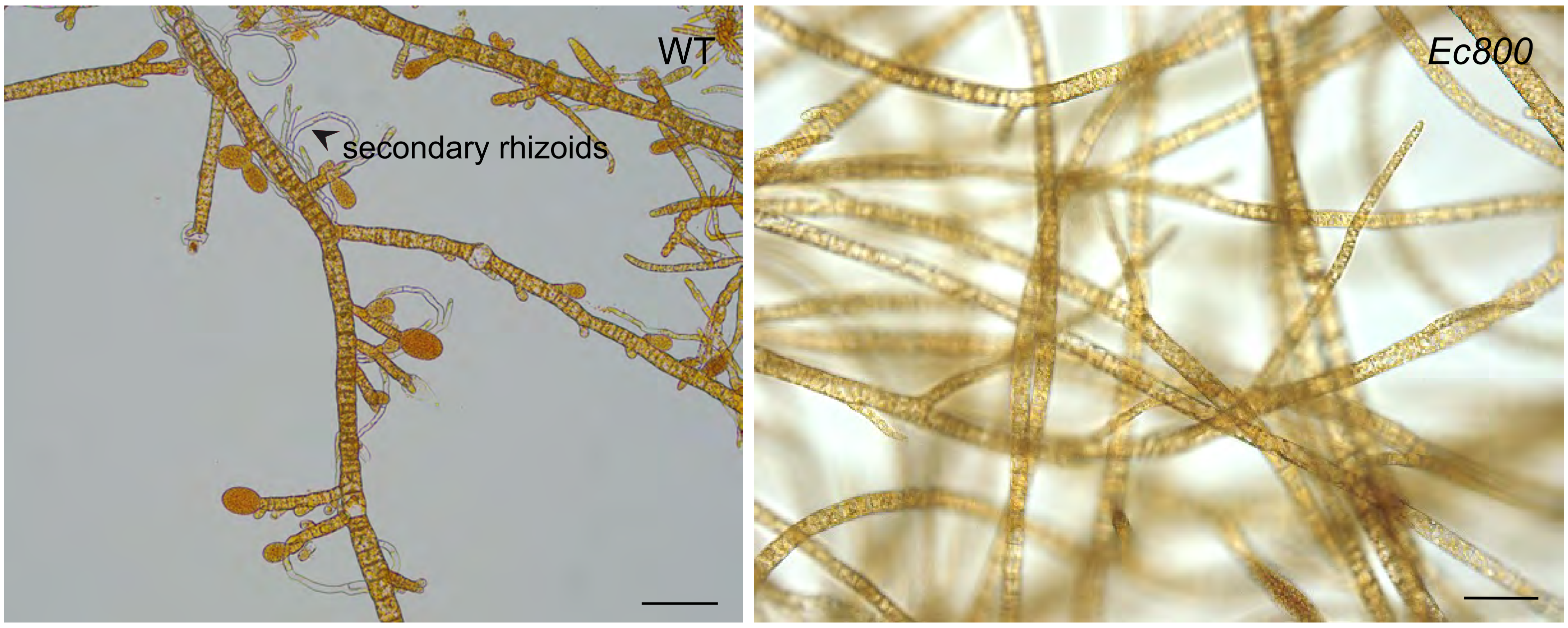

Fig. S1. Absence of secondary rhizoids in apical filaments of mutant lines compared with wild-type, after 3-weeks in culture. Scale=20 um.

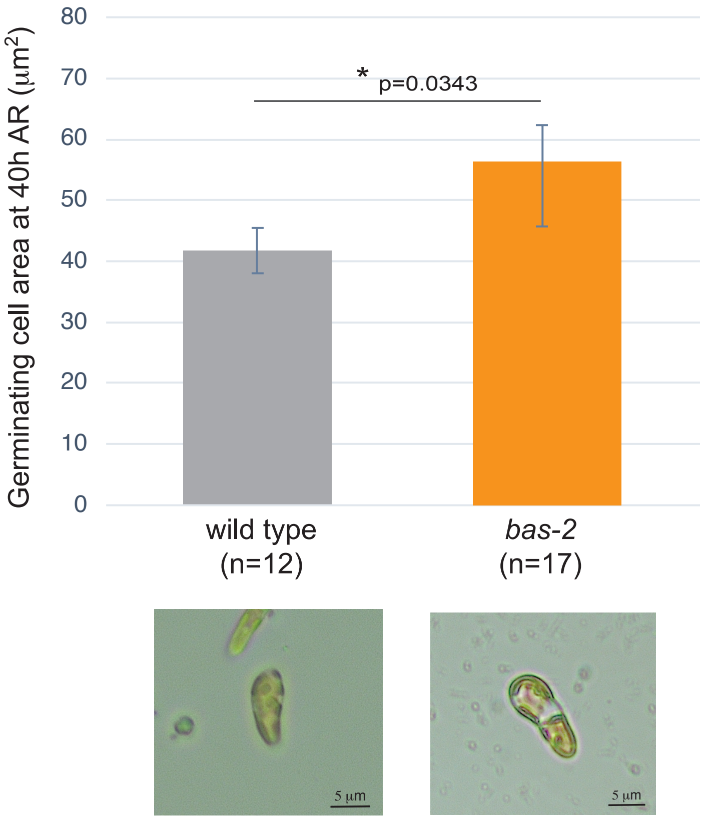

Fig. S2. Cell area in germinating initial cells of the wild-type versus *bas-2* mutants. Mea-surements of cell area (using Fiji) were performed 40 h after release (AR) on n = 19 germi-nating initial cells. Statistical differences were assessed using Mann-Whitney U test (p- value = 0.0343).

Development • Supplementary information

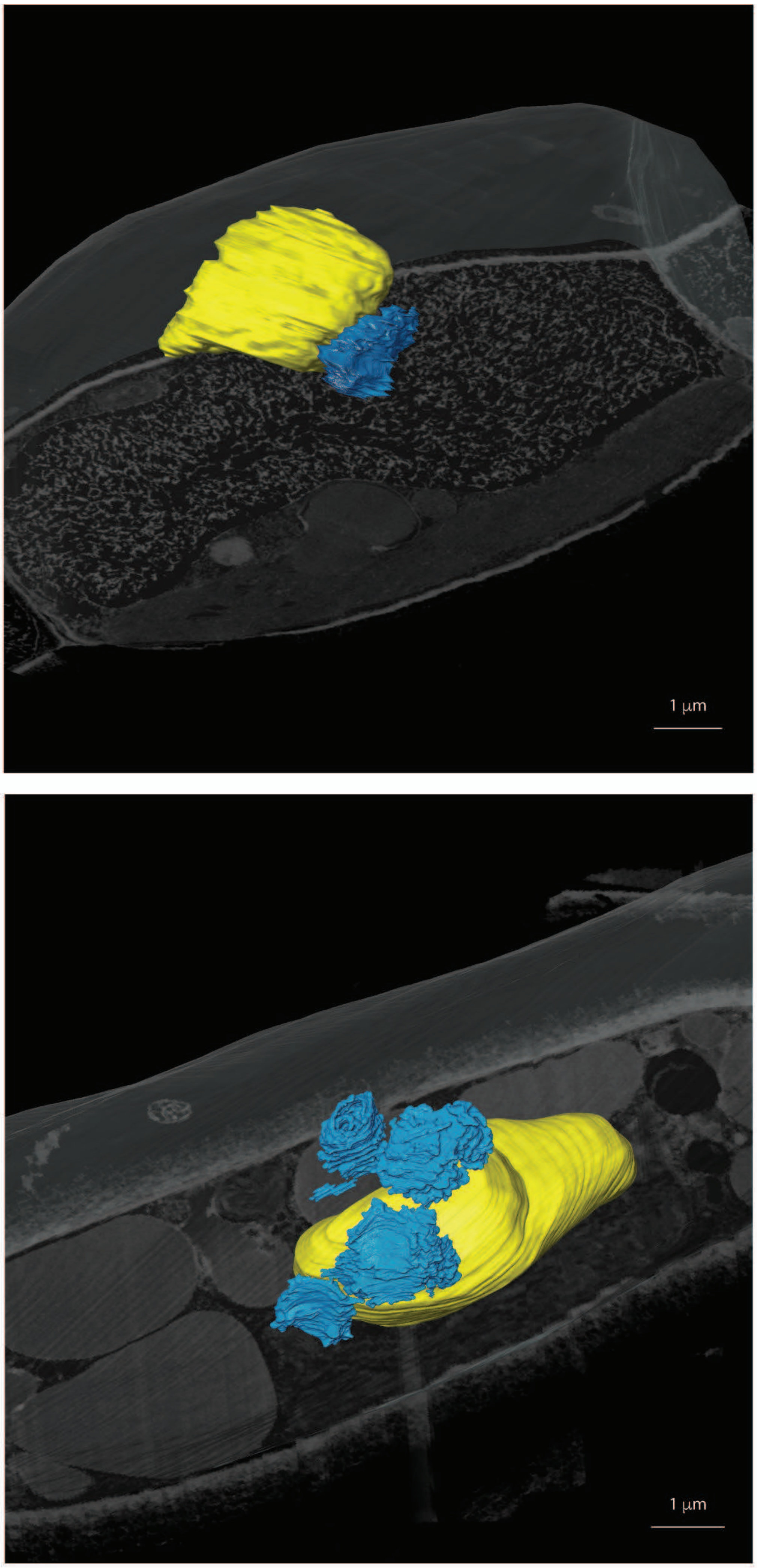

Fig. S3. 3D rendering of whole-cell FIB-SEM datasets showing a representative wild-type (WT) and *bas-2* mutant developing sporophytes. The Golgi complex (cyan),the nucleus (yellow) and the plasma membrane (grey) are shown. A slice from the raw image stack is shown in background.

Development • Supplementary information

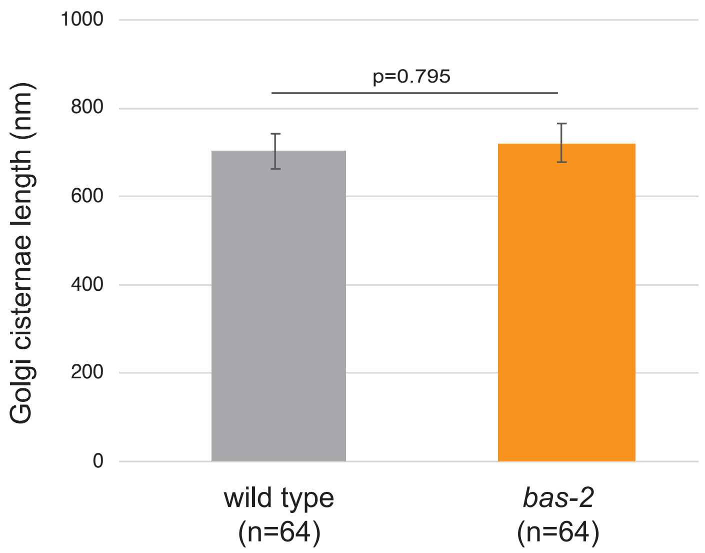

Fig. S4. Golgi cisternae length in wild-type and *bas-2* mutant cells.

Development • Supplementary information

A

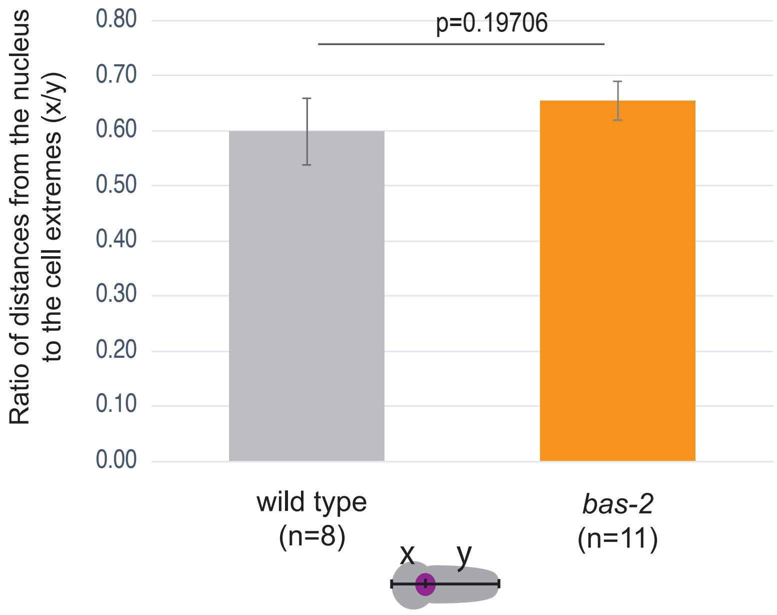

B

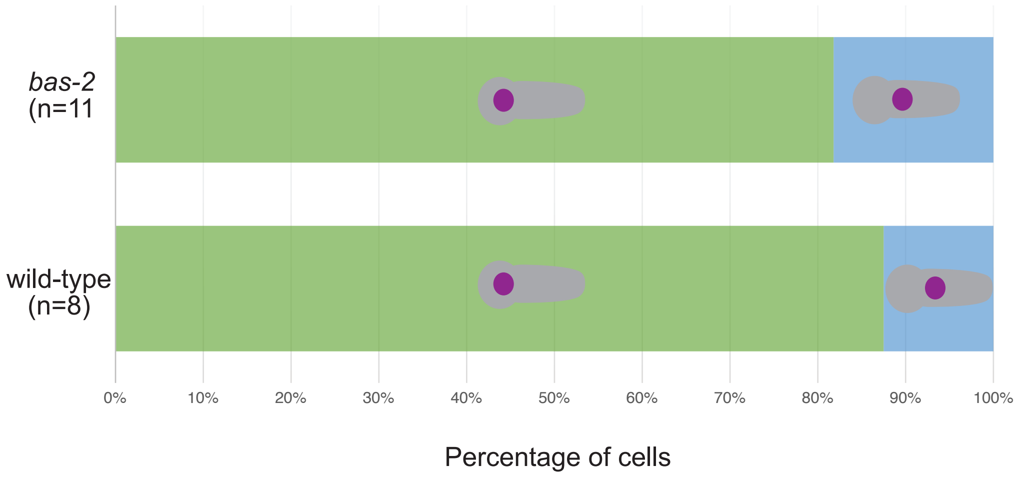

Fig. S5. (A) Relative positions of the nucleus in relation to the germination axis in wild-type and *bas-2* mutant initial cells. The number of cells scored is indicated in parentheses. There was no significant difference in the position of nucleus between wild-type and mutant cells (Mann-Whitney U Test, p= 0.19706. (B) Proportion of cells containing nuclei in central or posterior position in relation to the germination axis before the first cell division in wild-type and *bas-2* mutant initial cells. Nuclei in the mutant were not mispositioned compared with wild-type initial cells before the first cell divi-sion. The number of cells scored in each sample is indicated in parentheses.

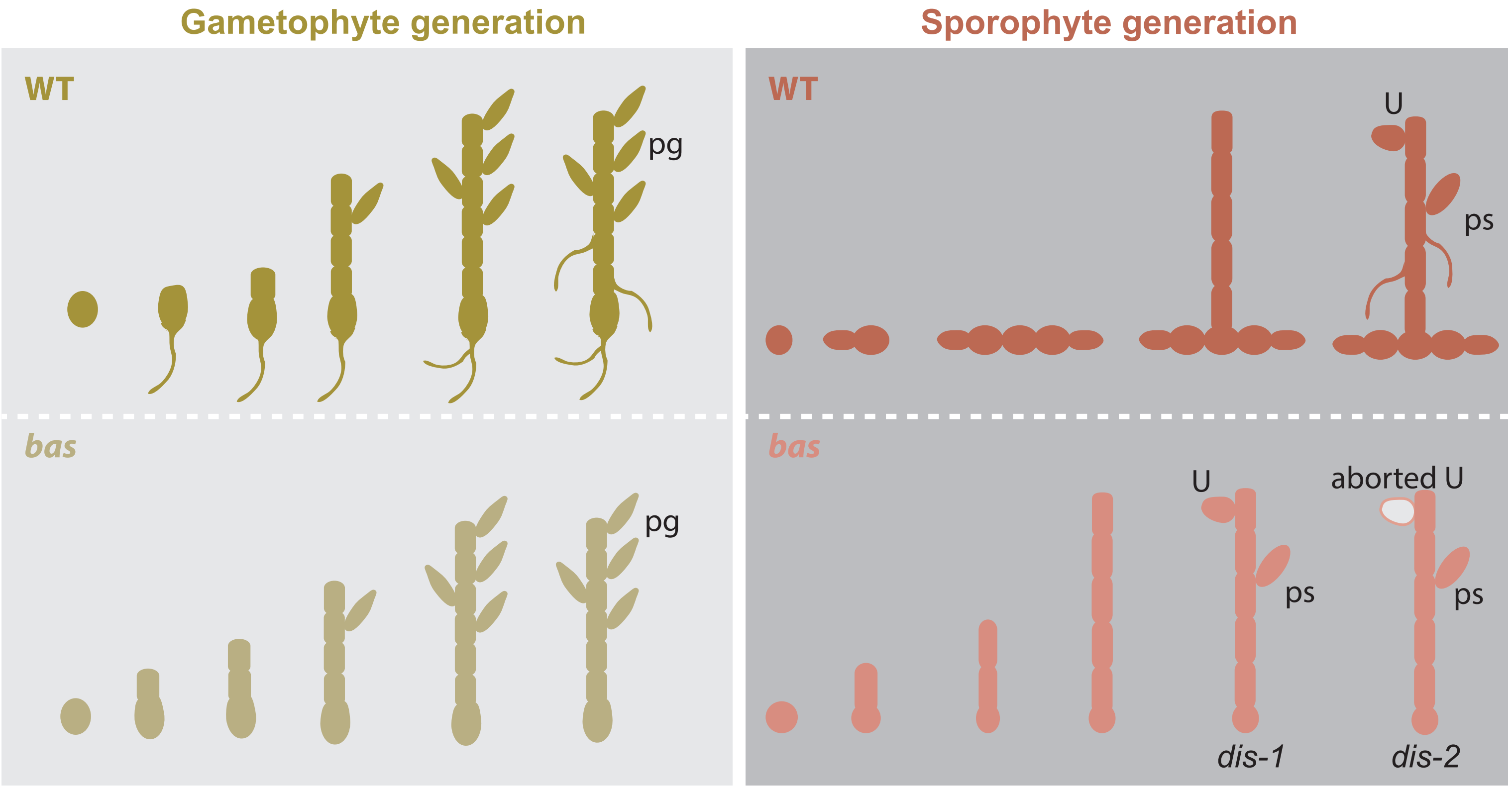

Fig. S6. Schematic view of the phenotype of wild type (WT) and *baseless* mutants. Note that *dis-2* gametophytes cannot be obtained because *dis-2* has a meiotic defect in the sporophyte generation.

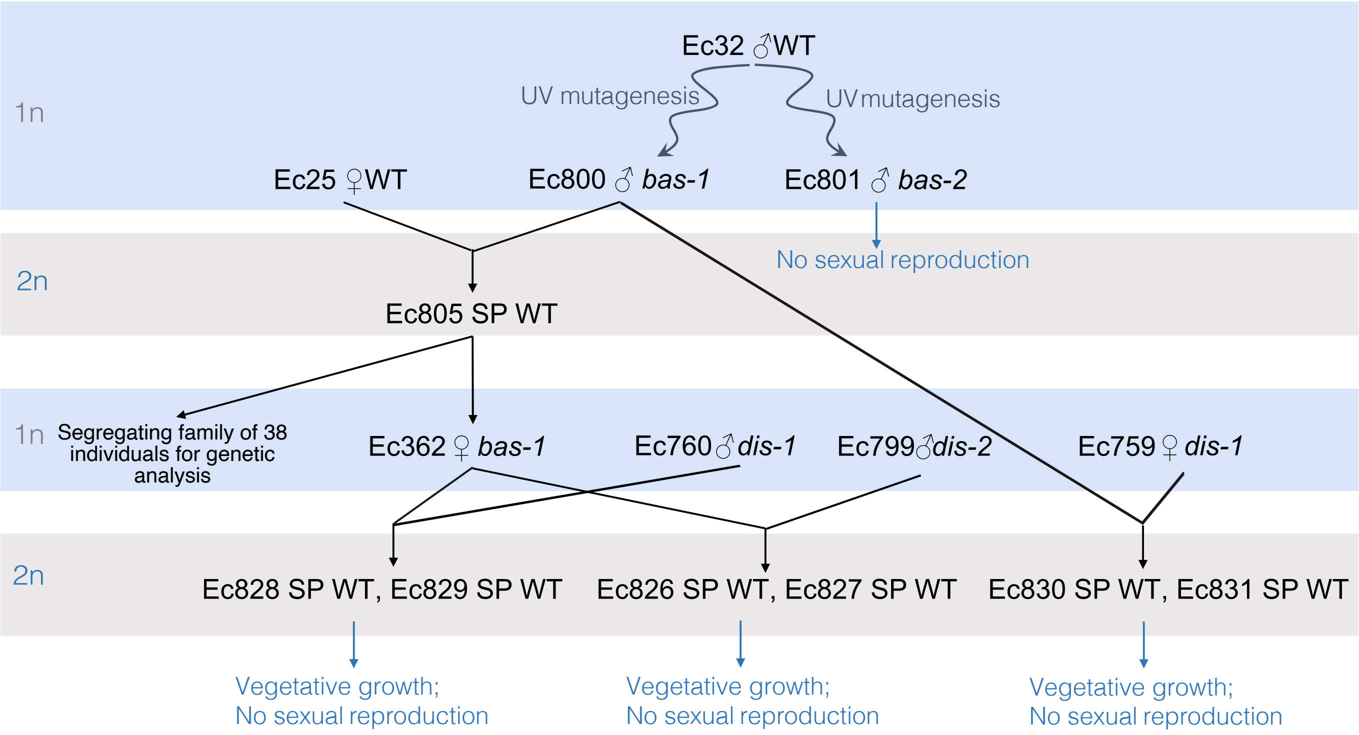

Fig. S7. Pedigree of the *Ectocarpus* strains used in this study. SP, diploid, hybrid sporophyte; WT, wild type; m, male; f, female.

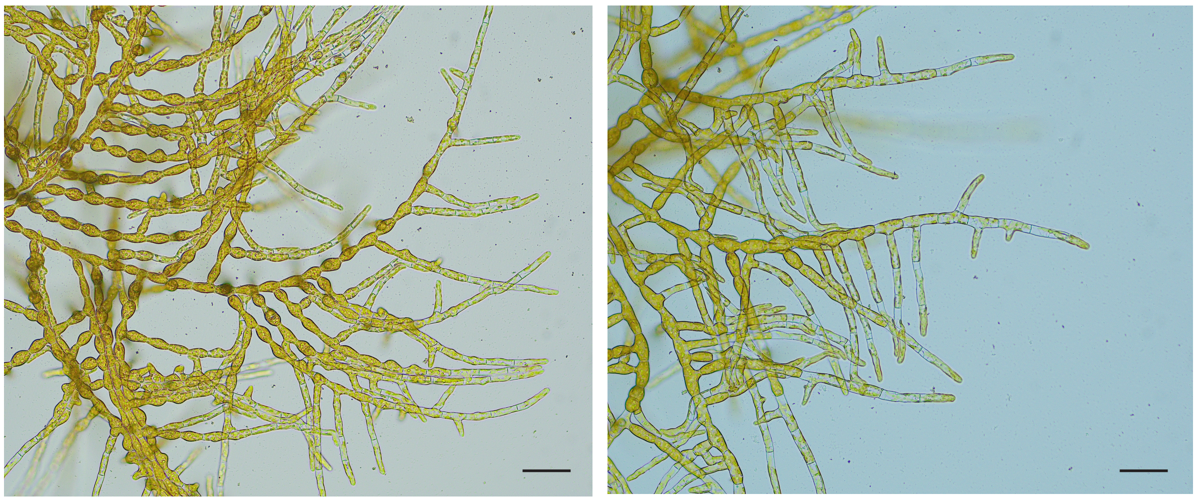

Fig. S8. Morphological phenotypes of diploid sporophytes derived by crossing *dis-2* x *bas-1* or *dis-1* x *bas-2*. Note that the diploid sporophyte derived from the cross has a wild-type phenotype, indicating the two mutations complement each other.

Development • Supplementary information

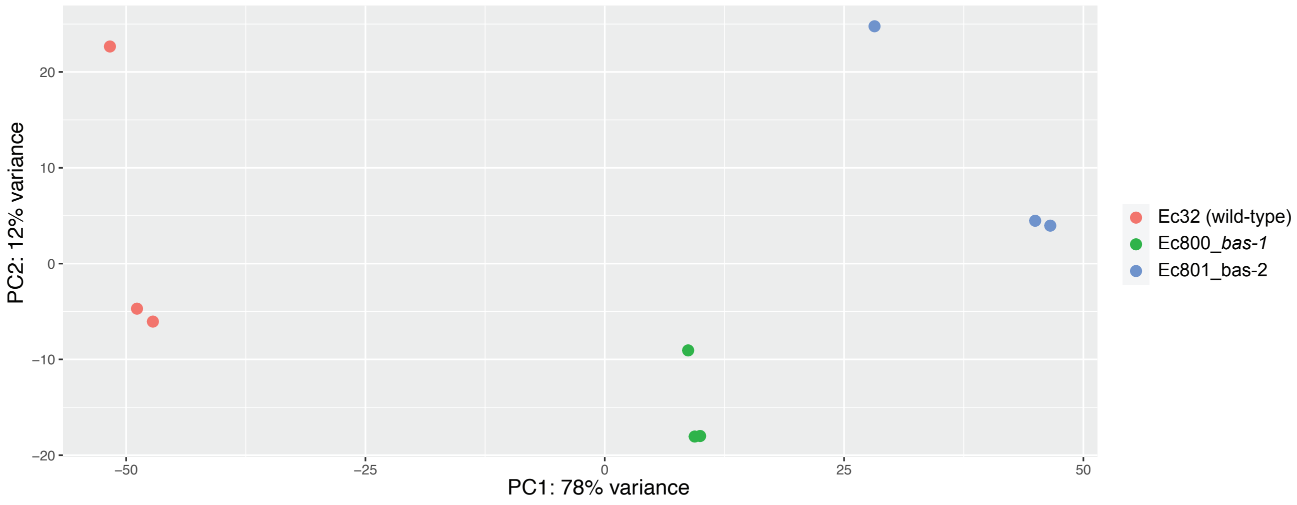

Fig. S9. Principal component analysis (PCA) comparison of transcript abundance patterns for all expressed genes across wild-type (WT), *bas-1* and *bas-2* replicate samples. The two dimensions represent 78% and 12% of the variance. The analysis was carried out using normalized counts generated by DESeq2 after Variance Stabiliz-ing Transformation (VST).

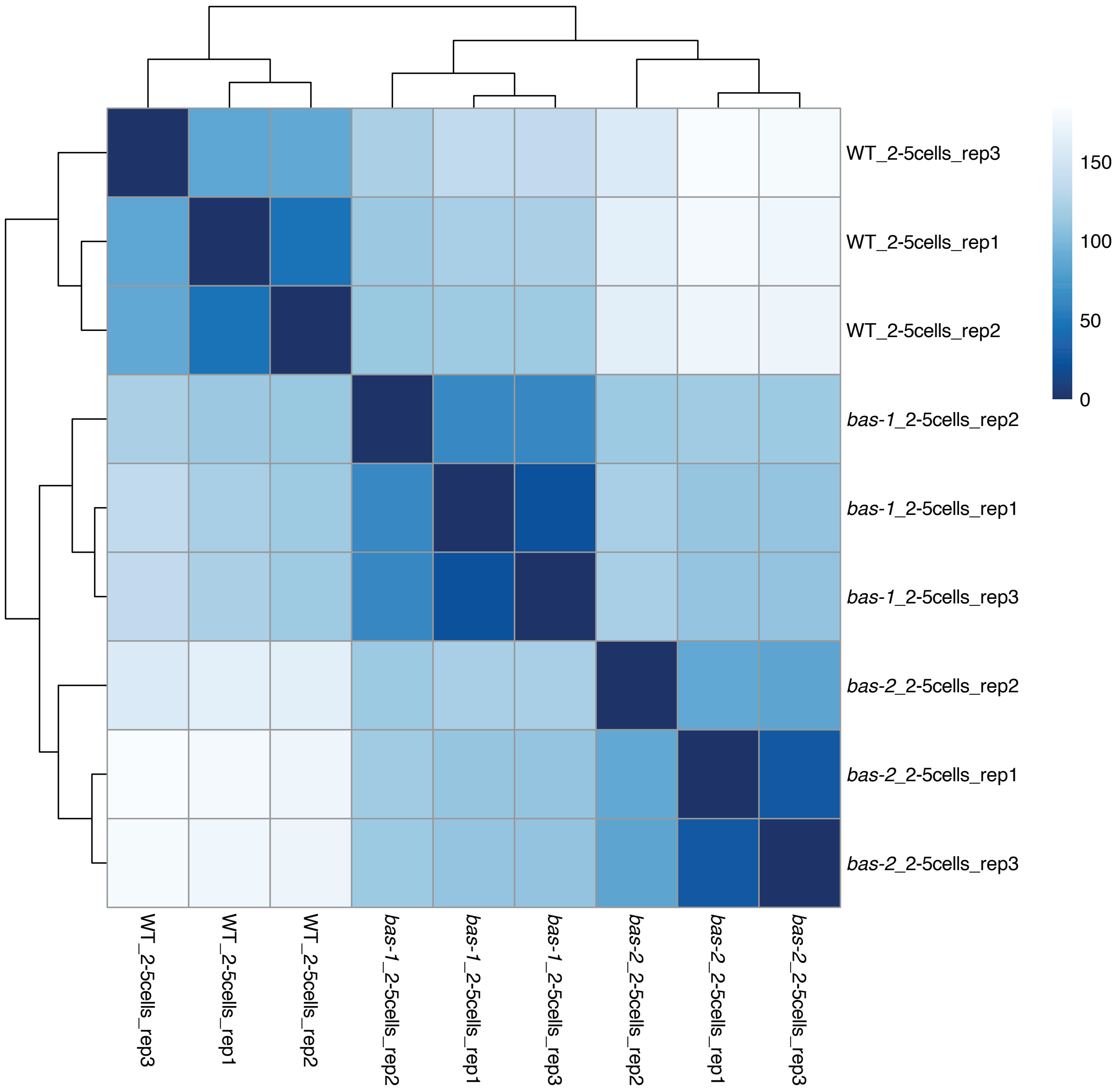

Fig. S10. Between-sample correlation diagnostics of the RNA sequencing data. Heat map representing the distance between replicates. Euclidian distances were calculating from normalized counts generated by DESeq2 after Variance Stabilizing Transformation (VST). Details of the samples are given in Table S7.

Development • Supplementary information

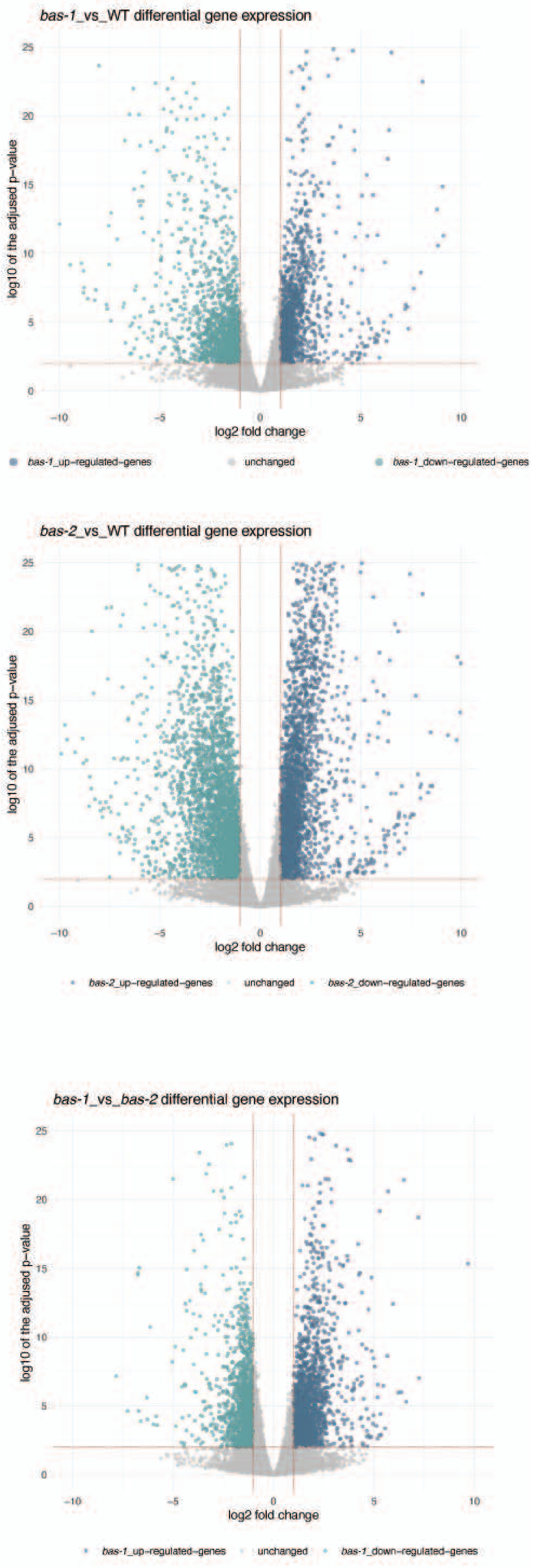

Fig. S11. Vulcano plots of all genes in pairwise comparisons between wild-type (WT) and mutants (bas-1 and bas-2).The log2 FC value was calculated based on the mean expression level (TPM) for each gene. Each dot represents one gene. Blue represents upregulated genes and green downregulated genes in each comparison.

Development • Supplementary information

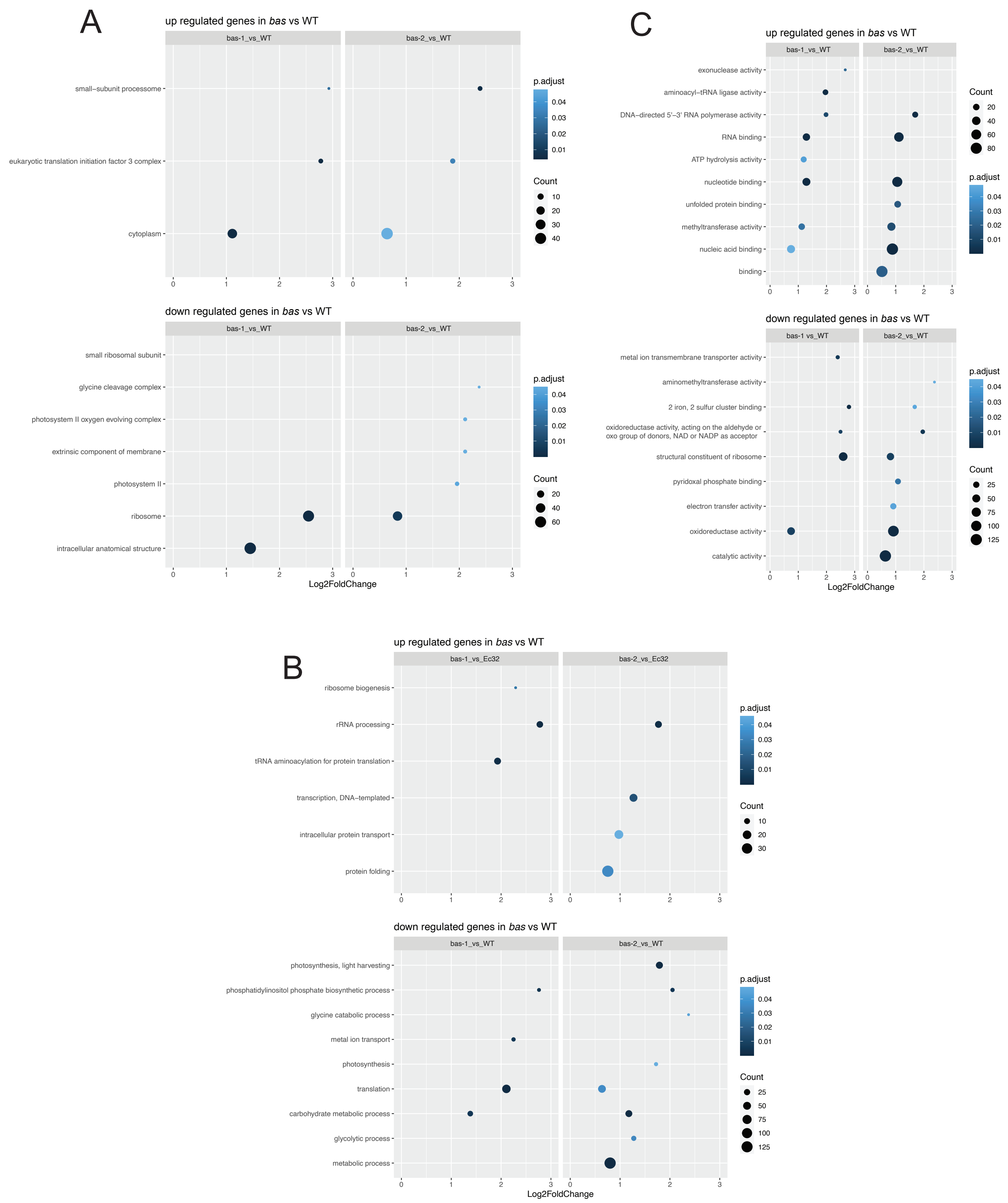

Fig. S12. GO term enrichment observed in DE gene sets in bas mutants compared to WT. Dot plot representation is divided according to GO term ontology classes 'Cellular Component' (A), 'Molecular Function' (B) and 'Biological Processes' (C).

Development • Supplementary information

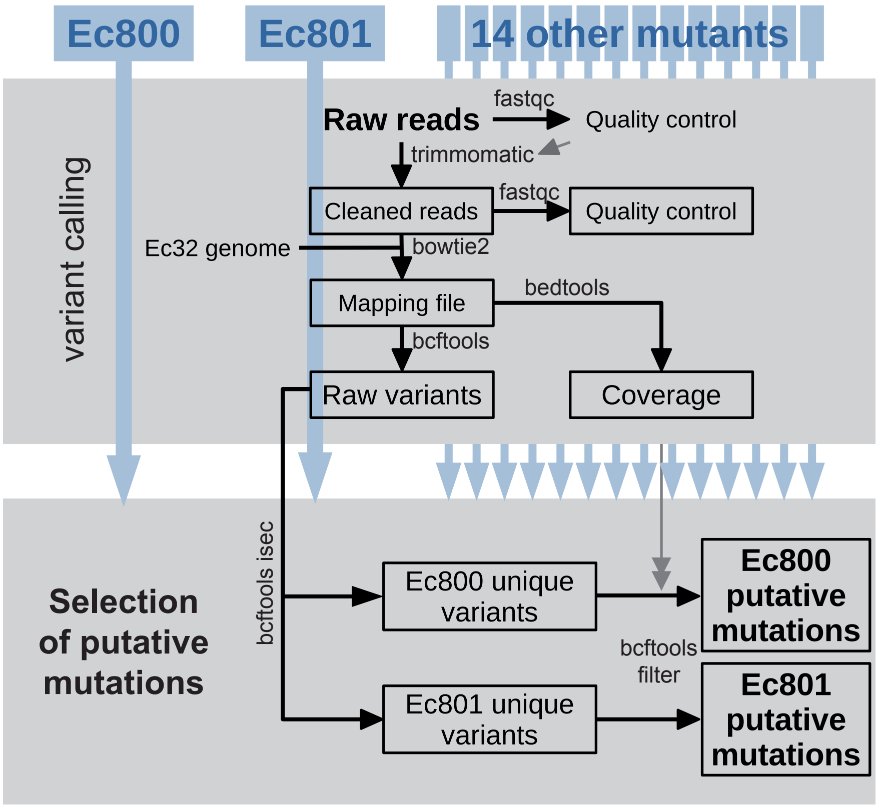

Fig. S13. Schematic diagram of the approach used to detect putative mutations in the genomes of Ec800 and Ec801.

Table S1. Strains used in this study. SP, sporophyte.

Click here to download Table S1

Table S2. Germination patterns in the wild type (wt) strain Ec32 compared with *bas-1* and *bas-2* initial cells.

Click here to download Table S2

Table S3. Measures of Golgi in wild type and bas-2 cells

Click here to download Table S3

Development • Supplementary information

Table S4. Phenotypic and genotypic analysis of the Ec805 segregating family. GA, gametophyte; B, baseless; WT, wild type; *bas*, *baseless*

Click here to download Table S4

Table S5. List of candidate mutations detected in the the genomes of the *bas-1* and *bas-2* mutants.

Click here to download Table S5

Table S6. Segregation of wild type and mutant alleles in bas progeny of a BAS (Ec25) x *bas* (Ec800) genetic cross. b, *bas*; wt, wild type.

Click here to download Table S6

Table S7. RNAseq libraries and sequencing datasets.

Click here to download Table S7

Table S8. Expression patterns of genes differentially expressed between samples at different FC

Click here to download Table S8

Table S9. Expression level of *Ectocarpus* sp7 genes in Transcripts Per kilobase Million (TPM) and DEseq2 results. young, 2-5 cell stage; SD, standard deviation.

Click here to download Table S9

Development • Supplementary information

Table S10. GO term enrichment analysis of the DE genes between the *baseless* mutant and wild-type.

Click here to download Table S10

Table S11. Sub-cellular localization enrichment analysis of the DE genes between the *baseless* mutant and wild-type. Significant enrichments are highlighted in bold.

Click here to download Table S11

Table S12. Functional classification and predicted cellular localisation of genes specifically activated or repressed in baseless mutants.

Click here to download Table S12

Table S13. Proportion of genes specifically activated or repressed in baseless mutants per functional category.

Click here to download Table S13

Table S14. Statistical analysis of BAS and DIS gene expression (linked to Fig 5).

Click here to download Table S14

Table S15. Statistical analysis of gene expression (linked to Fig 6B).

Click here to download Table S15
