# Supplementary material for: The baseless mutant links protein phosphatase 2A with basal cell identity in the brown alga Ectocarpus
Source: Development. 2023 Feb 14;150(4):dev201283. doi: 10.1242/dev.201283 (PMC10112911; doi:10.1242/dev.201283)
Supplement: Supplementary file 1 [file develop-150-201283_review_history.pdf]

The *baseless* mutant links protein phosphatase 2A with basal cell identity in the brown alga *Ectocarpus*

Olivier Godfroy, Min Zheng, Haiqin Yao, Agnes Henschen, Akira F. Peters, Delphine Scornet, Sebastien Colin, Paolo Ronchi, Katharina Hipp, Chikako Nagasato, Taizo Motomura, J. Mark Cock and Susana M. Coelho
DOI: 10.1242/dev.201283

Editor: Cassandra Extavour

Review timeline
Original submission: 15 September 2022
Editorial decision: 8 November 2022
First revision received: 22 December 2022
Accepted: 16 January 2023

Original submission

First decision letter

MS ID#: DEVELOP/2022/201283

MS TITLE: The baseless mutant links protein phosphatase 2A with basal cell identity in the brown alga Ectocarpus

AUTHORS: Olivier Godfroy, Min Zheng, Haiqin Yao, Agnes Henschen, Akira F Peters, Delphine Scornet, Sebastien Colin, Paolo Ronchi, Katharina Hipp, Chikako Nagasato, Taizo Motomura, J. Mark Cock, and Susana M. Coelho

I have now received all the referees' reports on the above manuscript, and have reached a decision. The referees' comments are appended below, or you can access them online: please go to BenchPress and click on the 'Manuscripts with Decisions' queue in the Author Area.

As you will see, the referees express considerable interest in your work, but have some significant criticisms and recommend a substantial revision of your manuscript before we can consider publication. The most important thing that I suggest you consider addressing if you choose to revise and resubmit, is whether and how the insights into mechanisms of development gained from the present analysis of the bas mutants goes above and beyond what was learned in your previously published analysis of the dis mutant in this system, as mentioned by Reviewer 2. If you are able to revise the manuscript along the lines suggested, which may involve further experiments, I will be happy receive a revised version of the manuscript. Your revised paper will be re-reviewed by one or more of the original referees, and acceptance of your manuscript will depend on your addressing satisfactorily the reviewers' major concerns. Please also note that Development will normally permit only one round of major revision. If it would be helpful, you are welcome to contact us to discuss your revision in greater detail. Please send us a point-by-point response indicating your plans for addressing the referee’s comments, and we will look over this and provide further guidance.

Please attend to all of the reviewers' comments and ensure that you clearly highlight all changes made in the revised manuscript. Please avoid using 'Tracked changes' in Word files as these are lost in PDF conversion. I should be grateful if you would also provide a point-by-point response detailing

© 2023. Published by The Company of Biologists under the terms of the Creative Commons Attribution License (https://creativecommons.org/licenses/by/4.0/).

how you have dealt with the points raised by the reviewers in the 'Response to Reviewers' box. If you do not agree with any of their criticisms or suggestions please explain clearly why this is so.

Reviewer 1

Advance summary and potential significance to field

This paper described the role of the BASELESS gene that is required for the development of basal system derived from the basal cell produced on division of the Ectocarpus spore in the haploid phase (gametophyte), and develops from the first two cells of the diploid phase (sporophyte). Evidence is presented that demonstrate that the gene acts to specify basal system development.

A forward genetic screen identified two recessive and putative loss of function alleles in the BASELESS gene. While germination in wild type partheno-sporophytes is bipolar – with cells emerging from opposite poles – the germination in baseless mutants is mainly unipolar. This is well described and an important phenotype suggesting that the development of one of the poles and perhaps the polarity of the spore or zygote is defective. The organization of microtubule arrays is defective during this early stage of development in the partheno-sporophyte. Ultrastructural analysis demonstrates that Golgi apparatus is morphologically defective but no other ultrastructural details were different from wild type.

BASELESS encodes a protein phosphatase 2A-related proteins and gene trees demonstrate its relationship to other conserved members of this class in other organisms. The gene is expressed at high levels in spores (consistent with its early function). Furthermore, expression of the DIS gene - which mutates to a similar phenotype – is lower in the baseless mutant, suggesting that BASELESS function is required for DIS expression.

Transcriptomes of baseless-1 and baseless-2 mutants were compared to each other and wild type. There are many differences in gene expression. The analysis identified 26 and 41 genes that are expressed in baselsss-1 and baseless -2 but not expressed in wild type. These genes may be responsible in part for the defective phenotypes in the mutants. The authors are careful not to overinterpret the results of this analysis, which is to be lauded.

The paper is well written and illustrated. The Abstract clear (I have made minor comments below). The Introduction is a very clear summary of the state of the art and places the work in the context of what is known about zygote development and axis formation in other photosynthetic organisms such as the brown alga Fucus and the flowering plants. It ends with a summary of what is known in Ectocarpus. The Results and Discussion are well written and clear.

It is an important contribution to a very important area of research.

Comments for the author

Minor comments

1. In the Abstract it would be good to make clear that the gametophyte is haploid and the sporophyte is diploid. At present it is unclear to a reader unfamiliar with organisms in which both haploid and diploid phases of the life cycle are multicellular.

2. In the Abstract would be useful if the gene names/protein names were spelled out in full in the abstract, for a reader from outside the field.

3. Results: The results are based on the phenotypes of two mutant alleles. If the authors have identified further alleles in BASELESS, it would be good to include them. While two alleles is sufficient to convince me the case is always stronger with more alleles. I know this might not be possible.

4. Results: Each section of the results would benefit from a clear statement of the conclusion of the data presented in that section.

© 2023. Published by The Company of Biologists under the terms of the Creative Commons Attribution License (https://creativecommons.org/licenses/by/4.0/).

Reviewer 2

Advance summary and potential significance to field

The authors report on the characterization of a new developmental mutant in the brown alga Ectocarpus, bas, which shows defects in producing basal structures that normally emerge in early development. Two alleles, bas-1 and bas-2 were identified in a mutagenized population. Both showed increased numbers of microtubule bundling and some evidence of increased golgi apparatus. Transcriptome analyses of early-stage development when polarity defects are emerging identified mis-regulated genes with some broad patterns of enrichment revealed by GO term enrichment. The bas locus encodes protein with similarity to a conserved subunit of type 2A eukaryotic protein phosphatases. The bas phenotype resembles that of a mutant, dis, that was previously described by the same group that also lacks basal structures and encodes a protein involved in microtubule organization. The data, analyses and writing are all very good, and I have only a few suggestions below. Many questions remain about how bas impacts development and how it interacts with dis. The findings certainly represent an advance, but much of what was described phenotypically was also seen previously in the dis mutant.

Comments for the author

1. I was unsure why only bas-1 was used for genetic analysis and not bas-2. The authors mentioned a sporophyte meiosis defect specific to bas-2, but that should not pose any problems in an outcross assuming that this is a recessive defect. The more severe defects in bas-2 were interpreted as likely being due to its more severe mutation than bas-1 (non-sense vs mis-sense), but this was not substantiated. My concern is that without an outcross and segregation analysis it cannot be ruled out that additional mutations in the bas-2 background contribute to its more severe defects or have other non-related impacts.

2. I was also curious why the authors did not try to isolate dis bas double mutant progeny after they did complementation experiments. The phenotypes of bas dis double mutants could be informative regarding additivity and pleiotropy, and could point to a shared pathway.

3. The cellular morphometrics in Figs. 3 and 4 look convincing, but I was wondering if the authors had checked cell size as a possible contributor to the increased microtubules and golgi? Also, in Fig. 3C there is not a clear metric on the y axis for microtubule bundles. It looks like per cell, but that should be explicitly stated.

4. Transcriptome analyses on developmental mutants with severe phenotypes such as bas or dis have limitations since a major structure is missing, and the mutations likely have pleiotropic effects as evidenced in the cytoskeletal and golgi defects. For example, it was surprising that bas mutants showed decreased representation of photosynthetic gene expression even though the missing basal/rhizoid tissue I assume would be less photosynthetic than the apical tissue that remains in the mutants. It would be helpful if the authors acknowledged the caveats of this analysis in the Results and/or Discussion.

5. In figure S4 there is a principal component plot of wild type and the two bas mutants. It looks like there is one sample in each of the genotypic replicates that is an outlier accounting for most of the variance in PC2. I wonder if the analysis would be more illuminating if those outlier samples were removed?

Reviewer 3

Advance summary and potential significance to field

In this manuscript, the authors describe the isolation, mutant phenotypes, and identification of the BASELESS (BAS) gene. In Ectocarpus, a brown algae, both the gametophyte and sporophyte develop from a single cell that is outside of the parental tissue. The first cell division of the gametophytic generation leads to an apical and basal cell that will form the “shoot” system or the rhizoid, respectively. In a UV mutagenesis, the authors isolated two alleles of the bas mutant. Subsequent genetic analysis indicated that bas-2 appeared to be the stronger allele. In either allele,

© 2023. Published by The Company of Biologists under the terms of the Creative Commons Attribution License (https://creativecommons.org/licenses/by/4.0/).

germination was typically unipolar, and a rhizoid system was not formed in either the gametophytic or sporophytic generation. This phenotype was reminiscient of the distag (dis) mutant which had been previously shown to be cause by a mutation in a TBCCd1 protein. Complementation tests between dis and bas-1 indicated these mutants were not allelic and crosses to a wild-type strain indicated bas-1 acted as a recessive mutation.

The authors then characterized the bas-1 and bas-2 alleles and found that they not only were defective in the production of rhizoids derived from the first cell division, but also could not make secondary rhizoids. Subsequent analysis of the microtubule cytoskeleton showed that bas-1 and bas-2 had more microtubule bundles than wild-type and found that the Golgi apparatus were about twice as numerous than in wild type although quantification of the later phenotype was not statistically significant.

The authors then cloned the BAS gene using whole genome sequencing and found only one CDS in both mutants that contained a deleterious base pair change. The BAS gene appears to encode a protein phosphate 2A regulatory B" subunit. Segregation analysis showed the mutation in bas-1 cosegregated with the bas-1 phenotype. The authors then conducted an RNA-seq experiment on both bas-1 and bas-2 compared to wildtype. Consistent with the bas-2 being a stronger alleles (caused by an early stop codon), they found gene expression changes, both up and down, were more extreme in the bas-2 allele. The BAS gene appears to be expressed throughout Ectocarpus development and is highly expressed in gametes. The DIS gene appears to be upregulated in the bas mutant backgrounds. Results from the RNA-seq experiment pointed to genes involved in photosynthesis and metabolism are downregulated in the bas mutants and genes involved in intracellular protein transport and protein synthesis were upregulated. The authors speculate that the later finding could provide a link to the defects seen in the Golgi apparatus.

Comments for the author

Overall, the manuscript was well written and easy to follow. The results are interesting, if not definitive and I agree with the authors that the study of Ectocarpus, which evolved multicellularity independently of plant and animal lineages, is very interesting. The manuscript is a bit thin on results and no definitive mechanism is identified, but it seems like work in this field is pretty young and these types of papers will provide a valuable starting point for other in the field. I only have a couple of comments/suggestions:

1-What happens to the basal cell in the bas mutant gametophytes? Does it just sit there and not divide or does it take on some sort of apical fate? Similarly, it is difficult for me to see from the pictures what the "basal" cells look like in the sporophytic generation (Figure 2). A more thorough description in the manuscript and perhaps a schematic of development in the mutant could help.

2-I am not sure how difficult it is to transform Ectocarpus, but a translational reporter for BAS would be very nice to see if it had any sort of asymmetric localization or specific organelle localization during the first cell division.

3-In the introduction and the discussion, the authors write about what is known about calcium asymmetries in Fucus during the first cell division and speculate a role for the BAS protein acting as a calcium sensor of some sort. Is it feasible to characterize wild-type Ectocarpus and the bas mutants with a calcium sensing dye during the first cell division? This may help to rule out that pathway or make a stronger connection to it.

4-In Fucus, directional light plays a role in this process. It would be good to mention if this is a known factor in Ectocarpus.

Minor editorial comments:

Figure 4-Scale bars for A-D would be helpful

Line 241-there is a z at the end of mutants

© 2023. Published by The Company of Biologists under the terms of the Creative Commons Attribution License (https://creativecommons.org/licenses/by/4.0/).

First revision

Author response to reviewers' comments

I have now received all the referees' reports on the above manuscript, and have reached a decision. The referees' comments are appended below, or you can access them online: please go to BenchPress and click on the 'Manuscripts with Decisions' queue in the Author Area.
As you will see, the referees express considerable interest in your work, but have some significant criticisms and recommend a substantial revision of your manuscript before we can consider publication. The most important thing that I suggest you consider addressing if you choose to revise and resubmit, is whether and how the insights into mechanisms of development gained from the present analysis of the bas mutants goes above and beyond what was learned in your previously published analysis of the dis mutant in this system, as mentioned by Reviewer 2.

Re: The *baseless* mutants, together with *dis* and in the future other mutants affected in their early development, are allowing us to gain insights into the pathways that lead to the asymmetrical first cell division and cell fate determination in this group of important multicellular organisms. The comparison with plant and animal models will hopefully shed light into the universality or uniqueness of the processes underlying early development across multicellular eukaryotes. We offer below (in reply to Reviewer 2) more details of why we believe the identification and characterisation of *baseless* mutants goes above and beyond our previous work with DIS.

If you are able to revise the manuscript along the lines suggested, which may involve further experiments, I will be happy receive a revised version of the manuscript. Your revised paper will be re-reviewed by one or more of the original referees, and acceptance of your manuscript will depend on your addressing satisfactorily the reviewers' major concerns. Please also note that Development will normally permit only one round of major revision. If it would be helpful, you are welcome to contact us to discuss your revision in greater detail. Please send us a point-by-point response indicating your plans for addressing the referee’s comments, and we will look over this and provide further guidance.

Re: Please find below a point by point response to the referees comments. In order to respond to the reviewers comments, we have now performed more phenotypic characterisation of the mutants, including more measurements of nuclear positions, cell area, Golgi fragmentation, together with a very thorough re-analysis of the transcriptomic datasets. The main conclusions of the paper are not changed, but we thank you and the reviewers for the interesting suggestions, which we believe have substantially enriched our paper.

Reviewer 1 Advance Summary and Potential Significance to Field:
This paper described the role of the BASELESS gene that is required for the development of basal system, derived from the basal cell produced on division of the Ectocarpus spore in the haploid phase (gametophyte), and develops from the first two cells of the diploid phase (sporophyte). Evidence is presented that demonstrate that the gene acts to specify basal system development.
A forward genetic screen identified two recessive and putative loss of function alleles in the BASELESS gene. While germination in wild type partheno-sporophytes is bipolar – with cells emerging from opposite poles – the germination in baseless mutants is mainly unipolar. This is well described and an important phenotype, suggesting that the development of one of the poles and perhaps the polarity of the spore or zygote is defective. The organization of microtubule arrays is defective during this early stage of development in the partheno- sporophyte. Ultrastructural analysis demonstrates that Golgi apparatus is morphologically defective but no other ultrastructural details were different from wild type.
BASELESS encodes a protein phosphatase 2A-related proteins and gene trees demonstrate its relationship to other conserved members of this class in other organisms. The gene is expressed at high levels in spores (consistent with its early function). Furthermore, expression of the DIS gene - which mutates to a similar phenotype – is lower in the baseless mutant, suggesting that BASELESS function is required for DIS expression.
Transcriptomes of baseless-1 and baseless-2 mutants were compared to each other and wild type. There are many differences in gene expression. The analysis identified 26 and 41 genes that are expressed in baselsss- 1 and baseless -2 but not expressed in wild type. These genes may be responsible in part for the defective phenotypes in the mutants. The authors are careful not to

© 2023. Published by The Company of Biologists under the terms of the Creative Commons Attribution License (https://creativecommons.org/licenses/by/4.0/).

overinterpret the results of this analysis, which is to be lauded.
The paper is well written and illustrated. The Abstract clear (I have made minor comments below). The Introduction is a very clear summary of the state of the art and places the work in the context of what is known about zygote development and axis formation in other photosynthetic organisms such as the brown alga Fucus and the flowering plants. It ends with a summary of what is known in Ectocarpus. The Results and Discussion are well written and clear.
It is an important contribution to a very important area of research.

Reviewer 1 Comments for the Author:
Minor comments

1. In the Abstract it would be good to make clear that the gametophyte is haploid and the sporophyte is diploid. At present it is unclear to a reader unfamiliar with organisms in which both haploid and diploid phases of the life cycle are multicellular.
Re: The reviewer is correct that classically we consider sporophytes to be diploid and gametophytes haploid. However, in many brown algae (and *Ectocarpus* in particular) life cycle generation may be decoupled from ploidy. For example, gametes that do not fuse with gametes of the opposite sex may develop parthenogenically to produce (haploid) sporophytes (as shown in Figure 1). Conversely, diploid gametophytes may be produced by polyploid sporophytes. We thought it would be too complicated to explain these ideas in the abstract, that is the reason why we have avoided stating strictly the ploidy for sporophytes and gametophytes in the abstract. We have added a section in the introduction to explain this in detail:

"Ectocarpus, as many brown algae, alternates between a gametophyte and a sporophyte generation, both being multicellular and independent (Figure 1). Male and female gametophytes produce male and female gametes by mitosis, which fuse to produce the (diploid) sporophyte. In absence of fusion with gametes of the opposite sex, germinating parthenogenetic gametes deploy the sporophyte program, despite being haploid, to produce partheno- sporophyte algae that are indistinguishable from diploid sporophytes in terms of morphology. Life cycle generation (i.e. deployment of a gametophyte or a sporophyte body plan) is therefore not determined by ploidy (haploid or diploid phase) and these two features of the life cycle can be uncoupled under certain circumstances (Bothwell et al., 2010a; Coelho et al., 2020). "

2. In the Abstract would be useful if the gene names/protein names were spelled out in full in the abstract, for a reader from outside the field.
Re: This has been done.

3. Results: The results are based on the phenotypes of two mutant alleles. If the authors have identified further alleles in BASELESS, it would be good to include them. While two alleles is sufficient to convince me, the case is always stronger with more alleles. I know this might not be possible.
Re: Unfortunately, we have not identified yet any further alleles of *baseless*.

4. Results: Each section of the results would benefit from a clear statement of the conclusion of the data presented in that section.
Re: This has been done.

Reviewer 2 Advance Summary and Potential Significance to Field:
The authors report on the characterization of a new developmental mutant in the brown alga Ectocarpus, bas, which shows defects in producing basal structures that normally emerge in early development. Two alleles, bas-1 and bas-2 were identified in a mutagenized population. Both showed increased numbers of microtubule bundling and some evidence of increased golgi apparatus. Transcriptome analyses of early-stage development when polarity defects are emerging identified mis-regulated genes with some broad patterns of enrichment revealed by GO term enrichment.
The bas locus encodes protein with similarity to a conserved subunit of type 2A eukaryotic protein phosphatases. The bas phenotype resembles that of a mutant, dis, that was previously described by the same group that also lacks basal structures and encodes a protein involved in microtubule organization. The data, analyses and writing are all very good, and I have only a few suggestions

© 2023. Published by The Company of Biologists under the terms of the Creative Commons Attribution License (https://creativecommons.org/licenses/by/4.0/).

below. Many questions remain about how bas impacts development and how it interacts with dis. The findings certainly represent an advance, but much of what was described phenotypically was also seen previously in the dis mutant.

Re: It is correct to say that that *distag* and *baseless* mutants have very similar phenotypes, although we note they are not fully identical, especially concerning the mis-positioning of the nucleus during germination (present in *distag* and not in *baseless* -please see new Figure S5), and the meiosis defect we observed in *bas*-2. Most importantly, the *bas* mutant provides the first demonstration for the role of a PP2A in cell fate determination in a multicellular organism outside plants and animals. Moreover, given the important role of the related (but not orthologous) FASS/TON2 protein in land plant development, the identification and characterisation of *bas* highlights the deep phylogenetic conservation of the role of phosphatase complex across kingdoms to determine cell identity during the first cell division. Finally, we would argue that *baseless* links important cell physiological observations in the model brown algal Fucus, in particular the critical role for Ca2+ intracellular signaling during early development, to genetic pathways involved in cell fate determination. In other words, the BASELESS protein allows us to propose the first link between physiological knowledge acquired on Fucus embryo decades ago and the genetic model Ectocarpus. Our manuscript also reveals the association between two members of the cell identity pathway, DIS and BAS, as a first step to fully disentangle the molecular basis of cell identity determination in brown algae. Much more work is ahead of us, and we have to catch up with the research in developmental biology achieved during the last 40 years for animal and plant multicellular models of development, but we believe that it is important to report these advances if we want to have a full view of Biology across the eukaryotic tree of life.

Reviewer 2 Comments for the Author:
1. I was unsure why only bas-1 was used for genetic analysis and not bas-2. The authors mentioned a sporophyte meiosis defect specific to bas-2, but that should not pose any problems in an outcross assuming that this is a recessive defect.

Re: *Ectocarpus* mutants are generated by mutagenesis of gametes and are recovered as (haploid) partheno- sporophytes after parthenogenetic germination. These partheno-sporophytes then go through a (apo)meiosis to produce gametophytes, which at fertility produce gametes by mitosis (please see Figure 1). The challenge is that *bas- 2* partheno-sporophytes did not produce gametophytes likely due to a meiotic defect, precluding the production of gametes needed for the backcross. We have provided a more detailed pedigree in new version of Supplemental Figure 1 and also a more thorough explanation of the life cycle.

The more severe defects in bas-2 were interpreted as likely being due to its more severe mutation than bas-1 (non- sense vs mis-sense), but this was not substantiated. My concern is that without an outcross and segregation analysis it cannot be ruled out that additional mutations in the bas-2 background contribute to its more severe defects or have other non-related impacts.

Re: We have indeed interpreted the more severe phenotype of the *bas*-2 as being due to a more severe mutation in the BAS gene, but the reviewer is correct that this effect could be due to other mutations due to the mutagenesis.

In the impossibility of backcrossing *bas-2*, we investigated if the more severe phenotype of *bas*-2 could be due to a higher number of mutations in the *bas*-2 background (“off-target mutations’). We found that *bas*-2 mutant had less mutations (65 in total, 14 in CDS) compared to the *bas-1* (118 total, 27 in CDS). In terms of type of mutation, the proportion was similar between the two mutants: 22.88% and 21.54% of the mutations affect CDS in *bas-1* and *bas*-2, respectively. Among those, silent mutations represent 40.74% in *bas-1* and 50% in *bas*-2, whereas 59.26% and 42.86% are missense mutations. The only non-sense mutation identified in *bas*-2 is the one affecting the *BASELESS* gene (Reviewer-Table 1 and 2).

In the *bas*-2 mutant, in addition to BASELESS, five other genes had a missense mutation but none had a function that could be associated to meiosis: one is a “Pectin lyase fold/virulence factor“, three are “hypothetical” or “conserved unknown protein” and the last gene encodes a “Dynein heavy chain” (Reviewer-Table 3). We ran InterProScan in order to identify functional domains on those proteins (Reviewer-Table 4). The Ec-02_001180.1 gene encode a ““Pectin lyase-like” protein

© 2023. Published by The Company of Biologists under the terms of the Creative Commons Attribution License (https://creativecommons.org/licenses/by/4.0/).

with 10 transmembrane domain; the mutation affect the SSF51126 superfamily and PTHR11319 (G PROTEIN-COUPLED RECEPTOR-RELATED) pattern. Multiple alignment with domains of proteins used to define those patterns show that the mutated amino-acid in *Ectocarpus* is not conserved indicating that this mutation is unlikely to have a strong effect on the protein function (Reviewer-Figure1). Mutation in gene Ec-06_007090.1 affect a PTHR36058, NUCLEOPHOSMIN, pattern. Sequence alignment shows that this pattern is highly conserve among terrestrial plants but clearly less conserved when considering other photosynthetic organisms or parasites from Excavata ; the amino-acid that is mutated in *bas-2* appears to be conserved among this subgroup. The S476L mutation affecting the Ec-16_001910.a gene, falls in a non-conserved region of a PTHR15131 snRNA-activating Protein Complex Subnuti 1. Finally, the "Dynein heavy chain" encoding gene Ec-19_002420.1 presents similarity with "Male Fertility Factor KL5" (PTHR46532) but the G2927E mutation does not affect a conserved amino-acid of this pattern. Inside this PTHR46532 pattern, InterProScan identified a SSF52540 "P-loop containing nucleoside triphosphate hydrolases" superfamily that overlap the "P-loop containing dynein motor region D4" PF12780 Pfam domain. Again, the G2927E mutation does not affect a conserved amino-acid of those functional domain.

In conclusion, it is unlikely that 'off target mutations' in the *bas-2* individuals contribute to the more severe phenotype and we privilege the hypothesis that the strong phenotype is caused by a severely disrupted BAS protein. We agree, however, that we cannot completely exclude the possibility that the 5 missense mutations have an impact in the *bas-2* phenotype, therefore we have added the following paragraph:

"Note that the more severe phenotype of bas-2 could be due to the higher number of background mutations compared with bas-1, and not a direct consequence of bas mutation per se. In order to test this, we investigated the genomes of bas-1 and bas-2 and compared the amount of "off-target" mutations. We found that bas-2 mutant had actually less mutations (65 in total, 14 in coding sequences, CDS) compared to the bas-1 (118 total, 27 in CDS) (Table S5). In terms of type of mutation, the proportion was similar between the two mutants: about 21-23% of the mutations affect CDS and among those 40-50% are silent, whereas 50-60% are missense. The only non-sense mutation that is identified in bas-2 is the one affecting the BAS locus. Five genes had a missense mutation but none had a function that could be associated to meiosis. Furthermore, the functional annotation of those five genes with InterProScan are not modified by the mutations, suggesting that none of those mutations directly affect a key amino- acid residue. In conclusion, it is unlikely that background mutations in the bas-2 individuals contribute to the more severe phenotype of this allele. "

2. I was also curious why the authors did not try to isolate dis bas double mutant progeny after they did complementation experiments. The phenotypes of bas dis double mutants could be informative regarding additivity and pleiotropy, and could point to a shared pathway.

Re: This is a very interesting point, unfortunately the diploid sporophyte carrying mutations in one allele of DISTAG and one allele of BASELESS did not produce unilocular sporangium, precluding any further genetic analysis. We therefore were never able to isolate dis-bas double mutant. This is in itself an interesting observation, and we thank the reviewer for pointing this out; we have added a sentence mentioning this result:

"Heterozygous sporophytes had a normal phenotype during vegetative growth, however none of the heterozygous sporophytes (Ec826, Ec827, Ec828, Ec829, Ec830 and Ec831) produce unilocular sporangia at maturity, suggesting a meiotic defect. Note that in Ec826, Ec827, Ec828, Ec829, bas was the female partner, while in Ec830 and Ec831 it was the male that was bas. Therefore, the sexual background of the mutant did not change the meiotic defect. This lack of meiosis in the heterozygous sporophyte precluded the analysis of any meiotic offspring to further investigate pleiotropy or additivity between DIS and BAS."

We have also included in the new version of the manuscript a more detailed pedigree that includes the information about consistent lack of meiosis in five independent sporophytes originating from crosses between different parents (new Figure S7, new Table S1).

3. The cellular morphometrics in Figs. 3 and 4 look convincing, but I was wondering if the authors had checked cell size as a possible contributor to the increased microtubules and golgi? Also, in Fig.

© 2023. Published by The Company of Biologists under the terms of the Creative Commons Attribution License (https://creativecommons.org/licenses/by/4.0/).

3C there is not a clear metric on the y axis for microtubule bundles. It looks like per cell, but that should be explicitly stated.

Re: We have now included in the manuscript the measurements of the cell area of wild-type versus *bas-2* germinating cells (using the same developmental stage as the one used for the microtubule imaging). We confirm that the mutant cells have a significantly larger area (mean area 42 um^2 in WT versus 58 um^2 in bas-2), and this is actually a reminiscent phenotype of the *distag* mutant. We cannot exclude that more numerous Golgi and higher number of microtubule bundles are associated with larger cells, although this feature is unlikely to explain the extensive disorganization of the microtubules in mutant cells. The new version of the manuscript includes a supplemental figure with the measures of cell area in mutant versus WT (new Figure S2), measurements of cisternae length (new Figure S4) and nuclear positioning in mutants versus WT (new Figure S5), and these additional phenotypes are also mentioned in the text.

We confirm that in Figure 3C the metrics is 'microtubule bundles per cell' and this information has been added in the figure.

4. Transcriptome analyses on developmental mutants with severe phenotypes such as bas or dis have limitations since a major structure is missing, and the mutations likely have pleiotropic effects as evidenced in the cytoskeletal and golgi defects. For example, it was surprising that bas mutants showed decreased representation of photosynthetic gene expression even though the missing basal/rhizoid tissue I assume would be less photosynthetic than the apical tissue that remains in the mutants. It would be helpful if the authors acknowledged the caveats of this analysis in the Results and/or Discussion.

Re: We fully agree with the reviewer that mutations at the BAS locus likely induce pleiotropic effects. This is actually one of the reasons why we restricted our transcriptomic analysis to the early developmental stage before the differentiation of the basal prostrate and upper erected filaments occurs in the wild-type in order to best tackle the direct effects of the mutation.

We have slightly modified the text in the results section to take in consideration the reviewer comment:

"Because of the severe impact of the bas mutations on the general thallus architecture at the adult stage (lack of basal structures) and the prominence of the bas phenotype during the early stages of development, we focused on the 2-5 cell stage during germination of the initial cell."

Concerning the results of the transcriptomic analysis, we understand the reviewer reservations but we would like to clarify some of the functions of rhizoids, basal and apical cells in Ectocarpus. While the rhizoids are indeed much less pigmented than other cell types in Ectocarpus, they do have chloroplasts therefore they can be considered 'photosynthetic' elements. Moreover, rhizoids in brown algae do not grow 'underground' (like in land plants) and are exposed to light. Importantly, *distag* mutants are not only devoid of rhizoids but also of basal cells – and these are particularly conspicuous in the sporophyte generation. Basal cells are heavily pigmented (please see Figure 2C and 2U), even more so than apical filaments. Therefore, it may not be that surprising that absence of basal system in the mutants is associated with downregulation of photosynthesis. Moreover, in Ectocarpus, the apical system is mainly dedicated to asexual and sexual reproduction (Figure 1). In sum, the down regulation of photosynthesis related genes (and in particular in bas-2) may therefore reflect both the impact of the mutation on the algae metabolism and/or reflect the lack of the basal cells which are probably heavily photosynthetic. In this respect we would not think there is necessarily a caveat in the transcriptomic analysis, but we agree that these considerations should be discussed. We have therefore added a sentence in the discussion.

"The down regulation of photosynthesis-related genes may reflect both the impact of the mutation on the algae photosynthetic metabolism but could also reflect the lack of the basal cells which are heavily photosynthetic. It is interesting to note that some of the intracellular protein transport transcription and protein synthesis functions can be linked with the Golgi apparatus, which appears to be affected by the bas mutation (…)"

© 2023. Published by The Company of Biologists under the terms of the Creative Commons Attribution License (https://creativecommons.org/licenses/by/4.0/).

5. In figure S4 there is a principal component plot of wild type and the two bas mutants. It looks like there is one sample in each of the genotypic replicates that is an outlier accounting for most of the variance in PC2. I wonder if the analysis would be more illuminating if those outlier samples were removed?

Re: We have re-run the analysis removing the "out-layers" replicates of each condition (WT_rep3, bas-1_rep2, bas- 2_rep3). We repeated all the analysis using the 2-replicate' option (presented as Reviewer-Figure1-3). In this 2-replicats principal component 1, that split samples according to genotype, explain 91% of the variance. Using this approach, we identified slightly more DEGs (29% more than when comparing *bas-1* with WT and 10% more in *bas-2* vs WT comparison). However, 90% of the genes significantly differentially expressed when considering the 3 replicates are also DE with the 2 replicates, suggesting that the 3-replicate analysis is robust. Genes that are DE in one analysis but not in the other are close to the padj and log2FC threshold used to define the significantly DEGs. Finally, having more DEGs does not modify the GO-term enrichment analysis.

In conclusion, removing those three "out-layer" replicates does not improve the analysis; on the contrary, we believe it reduced the statistical robustness and enlarge the DEGs set without bringing more functional information.

Note that the correlation analysis between replicates show that replicates cluster together with high confidence (Figure S10). Therefore, we are confident that the analysis is more informative and more robust if all replicates are included, and we would prefer to keep the original version.

Reviewer 3 Advance Summary and Potential Significance to Field:
In this manuscript, the authors describe the isolation, mutant phenotypes, and identification of the BASELESS (BAS) gene. In Ectocarpus, a brown algae, both the gametophyte and sporophyte develop from a single cell that is outside of the parental tissue. The first cell division of the gametophytic generation leads to an apical and basal cell that will form the "shoot" system or the rhizoid, respectively. In a UV mutagenesis, the authors isolated two alleles of the bas mutant. Subsequent genetic analysis indicated that bas-2 appeared to be the stronger allele. In either allele, germination was typically unipolar, and a rhizoid system was not formed in either the gametophytic or sporophytic generation. This phenotype was reminiscient of the distag (dis) mutant which had been previously shown to be cause by a mutation in a TBCCd1 protein. Complementation tests between dis and bas-1 indicated these mutants were not allelic and crosses to a wild-type strain indicated bas-1 acted as a recessive mutation.

The authors then characterized the bas-1 and bas-2 alleles and found that they not only were defective in the production of rhizoids derived from the first cell division, but also could not make secondary rhizoids. Subsequent analysis of the microtubule cytoskeleton showed that bas-1 and bas-2 had more microtubule bundles than wild-type and found that the Golgi apparatus were about twice as numerous than in wild type, although quantification of the later phenotype was not statistically significant.

The authors then cloned the BAS gene using whole genome sequencing and found only one CDS in both mutants that contained a deleterious base pair change. The BAS gene appears to encode a protein phosphate 2A regulatory B" subunit. Segregation analysis showed the mutation in bas-1 cosegregated with the bas-1 phenotype. The authors then conducted an RNA-seq experiment on both bas-1 and bas-2 compared to wildtype. Consistent with the bas-2 being a stronger alleles (caused by an early stop codon), they found gene expression changes, both up and down, were more extreme in the bas-2 allele. The BAS gene appears to be expressed throughout Ectocarpus development and is highly expressed in gametes. The DIS gene appears to be upregulated in the bas mutant backgrounds. Results from the RNA-seq experiment pointed to genes involved in photosynthesis and metabolism are downregulated in the bas mutants and genes involved in intracellular protein transport and protein synthesis were upregulated. The authors speculate that the later finding could provide a link to the defects seen in the Golgi apparatus.

Reviewer 3 Comments for the Author:
Overall, the manuscript was well written and easy to follow. The results are interesting, if not definitive and I agree with the authors that the study of Ectocarpus, which evolved multicellularity independently of plant and animal lineages, is very interesting. The manuscript is a bit thin on

© 2023. Published by The Company of Biologists under the terms of the Creative Commons Attribution License (https://creativecommons.org/licenses/by/4.0/).

results and no definitive mechanism is identified, but it seems like work in this field is pretty young and these types of papers will provide a valuable starting point for other in the field. I only have a couple of comments/suggestions:

1-What happens to the basal cell in the bas mutant gametophytes? Does it just sit there and not divide or does it take on some sort of apical fate? Similarly, it is difficult for me to see from the pictures what the "basal" cells look like in the sporophytic generation (Figure 2). A more thorough description in the manuscript and perhaps a schematic of development in the mutant could help.

Re: The initial cell of the gametophyte generation in the *bas* mutant germinates and the first cell division produces two apical cells (Figure 2). This description is highlighted in the sentence: *"Initial cells of Ec800 and Ec801 gametophytes immediately developed as apical upright filament cells and no rhizoid cells were produced.* "

So, no rhizoids are produced in the *bas* gametophyte generation, so similarly to distag, mutations in *bas* loci disturb basal cell formation in both generations. All cells arising from the initial cell of the gametophyte generation are apical cells. Note however that (similarly to *distag*) *bas* partheno-sporophytes occasionally produced one or more enlarged and abnormally shaped cells (described in Figure 2) at the end where the second germ tube would normally emerge, possibly corresponding to an aborted basal system.

As suggested by the reviewer, we have included a further supplemental figure with the scheme of the development of wild type and mutants to clarify (new Figure S6). Further photos of early germinating initial cells are provided in new Figure S2 (to illustrate the cell area differences between mutants and WT).

2-I am not sure how difficult it is to transform Ectocarpus, but a translational reporter for BAS would be very nice to see if it had any sort of asymmetric localization or specific organelle localization during the first cell division.

Re: We thank the reviewer for the interesting suggestion. Unfortunately, while CRISPR-Cas9 approaches to know out genes are recently available for *Ectocarpus* (Badis et al. New Phytol. 2021) it is not yet possible to transform this organism. Therefore, the experiment proposed by the reviewer is currently not feasible.

3- In the introduction and the discussion, the authors write about what is known about calcium asymmetries in Fucus during the first cell division and speculate a role for the BAS protein acting as a calcium sensor of some sort. Is it feasible to characterize wild-type Ectocarpus and the bas mutants with a calcium sensing dye during the first cell division? This may help to rule out that pathway or make a stronger connection to it.

Re: We thank the reviewer again for the excellent idea. In the brown alga Fucus, intracellular Ca^{2+} experiments have been performed to follow Ca^{2+} changes during embryogenesis and in response to stimuli (e.g. osmotic stress). However, the introduction of Ca^{2+} dyes into the cells requires microinjection of dyes (because of the thick cell wall in brown algae). While this is possible to do in Fucus, which has large zygotes (100 micron) this task is a not trivial in the tiny cells of *Ectocarpus* (less than 5 micron for the initial cells). The other possibility would be transformation with a Ca^{2+} reporter, but as described above, transformation protocols are not yet available for this emerging model organism.

4- In Fucus, directional light plays a role in this process. It would be good to mention if this is a known factor in Ectocarpus.

Re: We assume the process that the reviewer is referring to is the effect of light in polar axis determination via Ca^{2+}? This is indeed what is believed to happen in Fucus, and considering that Ectocarpus initial cells also respond to light vectors (both the sporophyte and gametophyte generations germinate in a negatively phototropic manner but the response is less marked in the sporophyte generation), than we would assume similar processes are involved. There are no studies directly addressing the link between Ca2+ and polar axis determination in Ectocarpus (due to the technical challenges described above).

© 2023. Published by The Company of Biologists under the terms of the Creative Commons Attribution License (https://creativecommons.org/licenses/by/4.0/).

Minor editorial comments:

Figure 4-Scale bars for A-D would be helpful

Re: The scale bars were shown in the original figure (bottom right in each panel) and the legend states “Scale bar=200 nm”).

Line 241-there is a z at the end of mutants

Re: this has been corrected.

Ec-02_001180.1	PANTHER	PTHR11319	G PROTEIN-COUPLED RECEPTOR-RELATED	550	644	Glycine 593 → Glutamic acid (G593E)
	SUPERFAMILY	SSF51126	Pectin lyase-like	557	848	

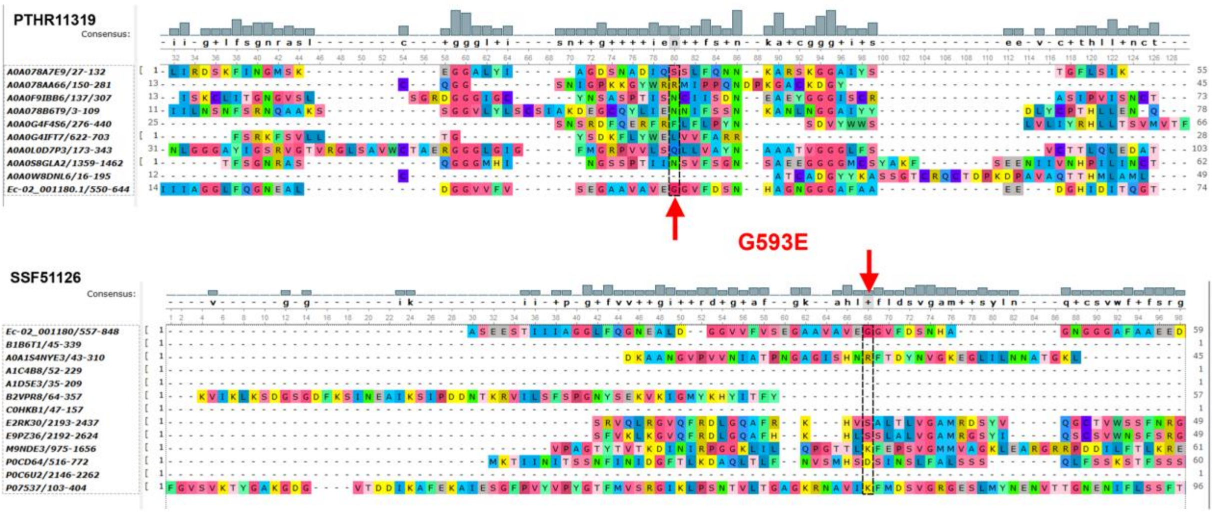

Ec-06_007090.1	PANTHER	PTHR36058:SF1	NUCLEOPHOSMIN	4	223	Histidine 67 → tyrosine (H67Y)

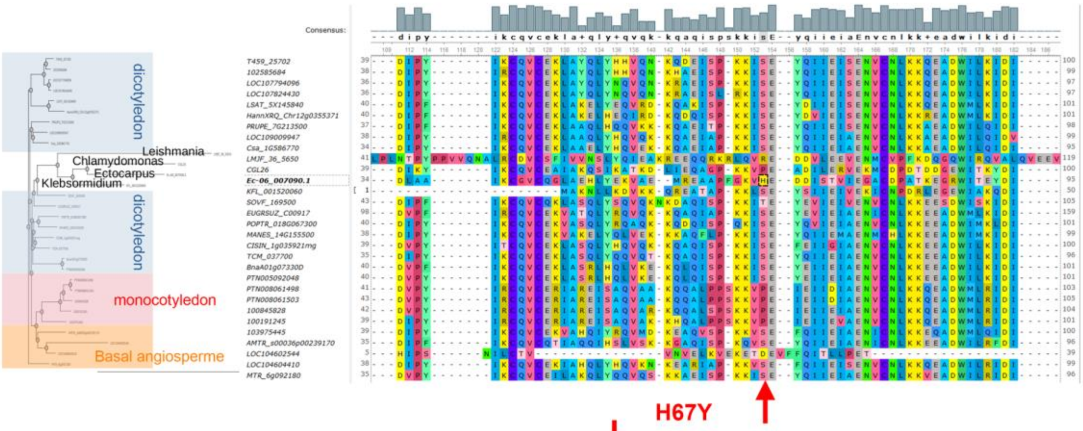

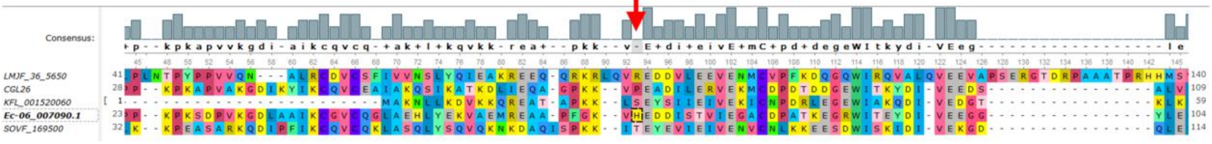

© 2023. Published by The Company of Biologists under the terms of the Creative Commons Attribution License (https://creativecommons.org/licenses/by/4.0/).

Ec-16_001910.1	PANTHER	PTHR15131:SF3	SNRNA-ACTIVATING PROTEIN COMPLEX SUBUNIT 1	42	805	Serine 476 → Leucine (S476L)

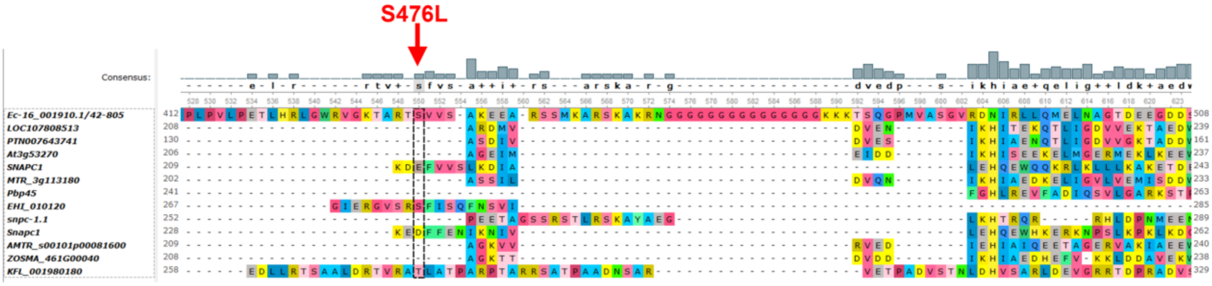

Ec-19_002420.1	PANTHER	PTHR46532:SF5	MALE FERTILITY FACTOR KL5	14	4509	Glycine 2927 → Glutamic acid (G2927E)
	SUPERFAMILY	SSF52540	P-loop containing nucleoside triphosphate hydrolases	2799	3071	
	Pfam	PF12780	P-loop containing dynein motor region D4	2803	3061	

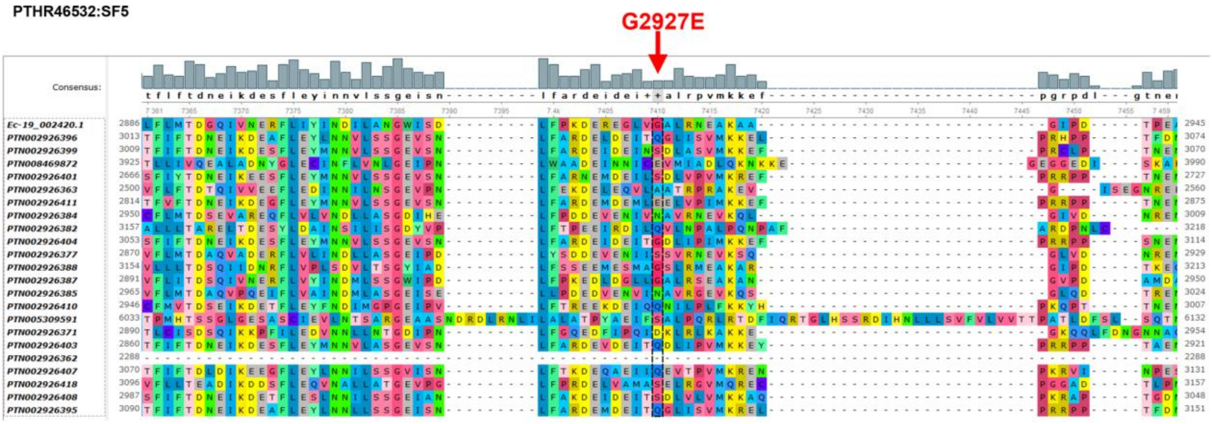

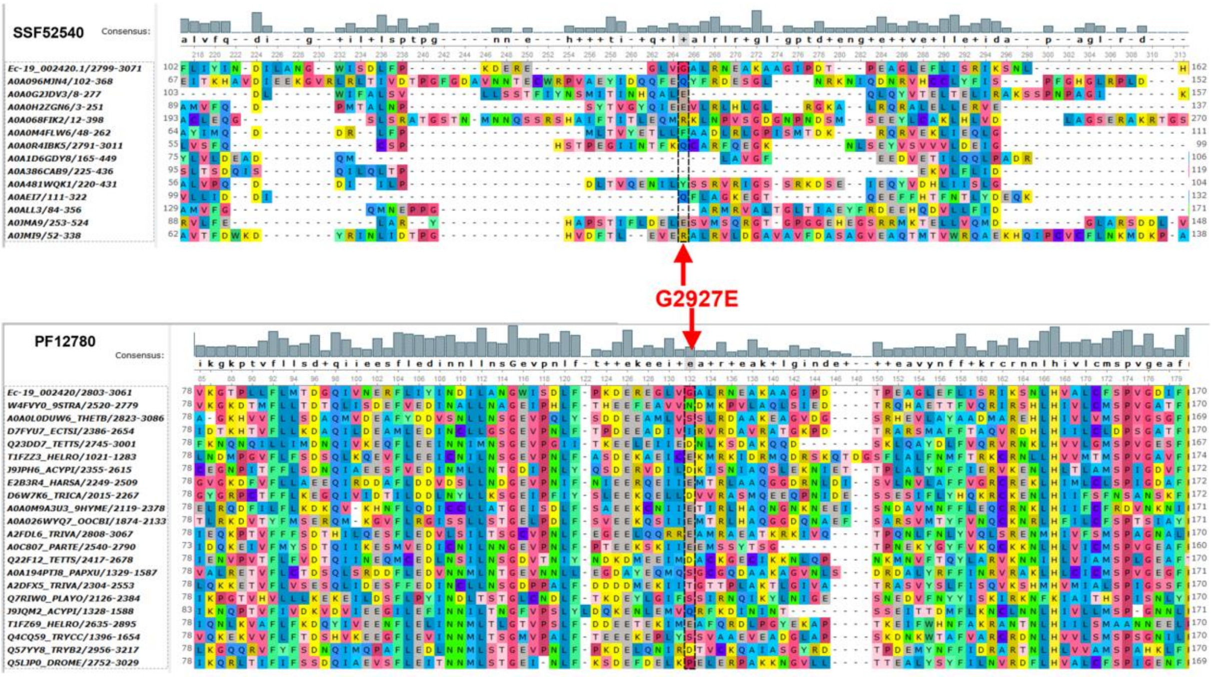

Reviewers-Figure 1. 2-replicats Differential gene expression analysis

A) equivalent to Fig S4. Principal component analysis (PCA) comparison of transcript abundance patterns for all expressed genes across wild-type (WT), *bas-1* and *bas-2* replicate samples. The two dimensions represent 91% and 8% of the variance. The analysis was carried out using normalized counts generated by DESeq2 after Variance Stabilizing Transformation (VST).

© 2023. Published by The Company of Biologists under the terms of the Creative Commons Attribution License (https://creativecommons.org/licenses/by/4.0/).

B) equivalent to Fig S5. Between-sample correlation diagnostics of the RNA sequencing data. Heat map representing the distance between replicates. Euclidian distances were calculating from normalized counts generated by DESeq2 after Variance Stabilizing Transformation (VST). Details of the samples are given in Table S7.

C) equivalent to FigS6. Vulcano plots of all genes in pairwise comparisons between wild-type (Ec32) and mutants (*bas-1* = *Ec800* and *bas-2* = *Ec801*). The log2 FC value was calculated based on the mean expression level (TPM) for each gene. Each dot represents one gene. Blue represents upregulated genes and green downregulated genes in each comparison.

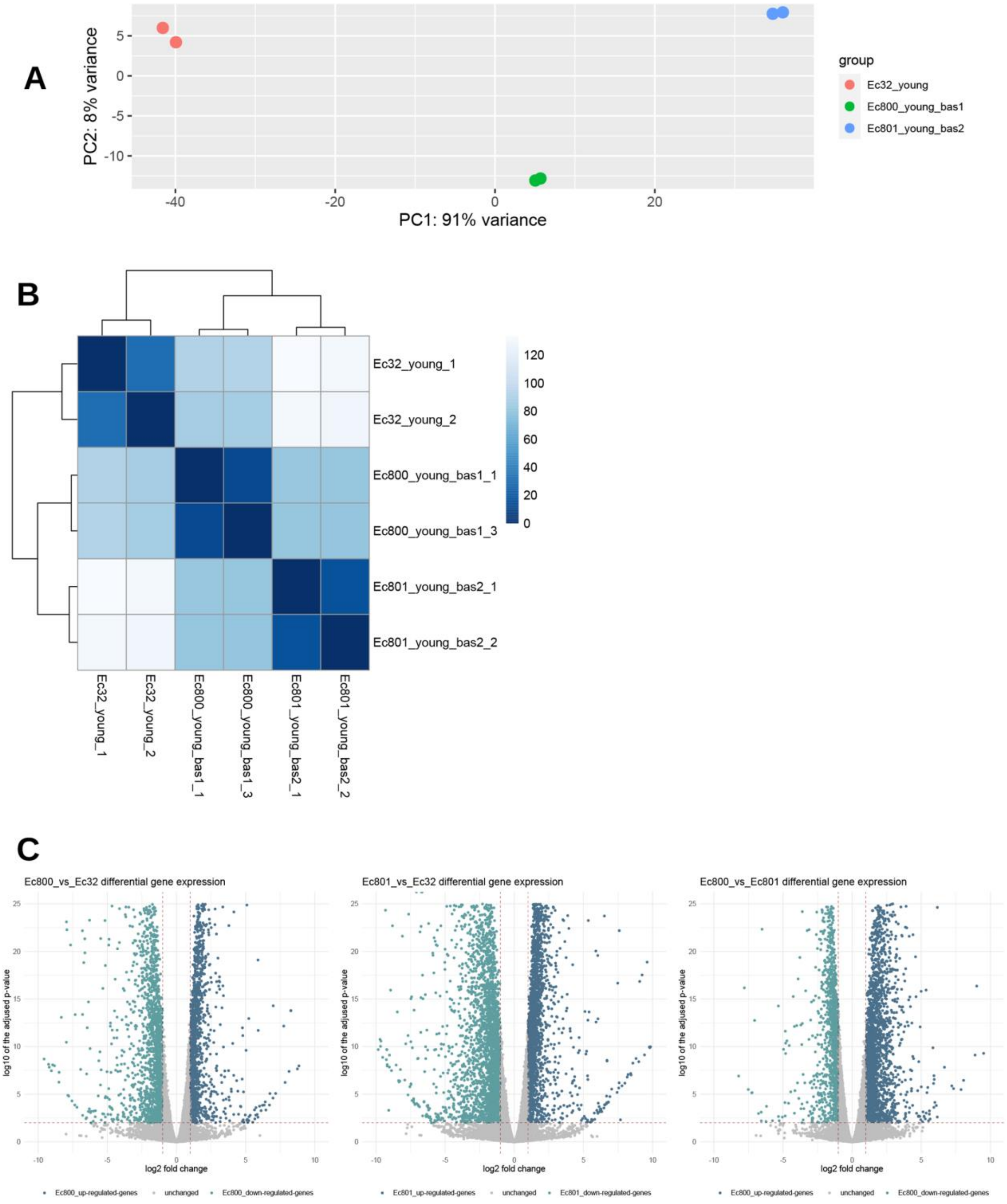

D) equivalent to Fig7. Differential gene expression analysis. **a**) Venn diagram of intersects of DE gene sets in bas mutants compare to wild-type. **b**) Boxplot representation of the distribution of gene expression levels (in log2 of TPM values *+1) of the DE gene sets from comparisons of either*

© 2023. Published by The Company of Biologists under the terms of the Creative Commons Attribution License (https://creativecommons.org/licenses/by/4.0/).

bas-1 (Ec800) *or* bas-2 (Ec801) *with wild-type (Ec32).* ***c-d****) Histogram representation of the distribution of fold changes of the DE gene sets from comparisons of bas-1 or bas-2 with WT; vertical dashed bars indicate the medians of the distributions. DE genes in bas-1 compared to wild-type (****c****); DE genes in bas-2 compared to wild-type (****d****).*

D

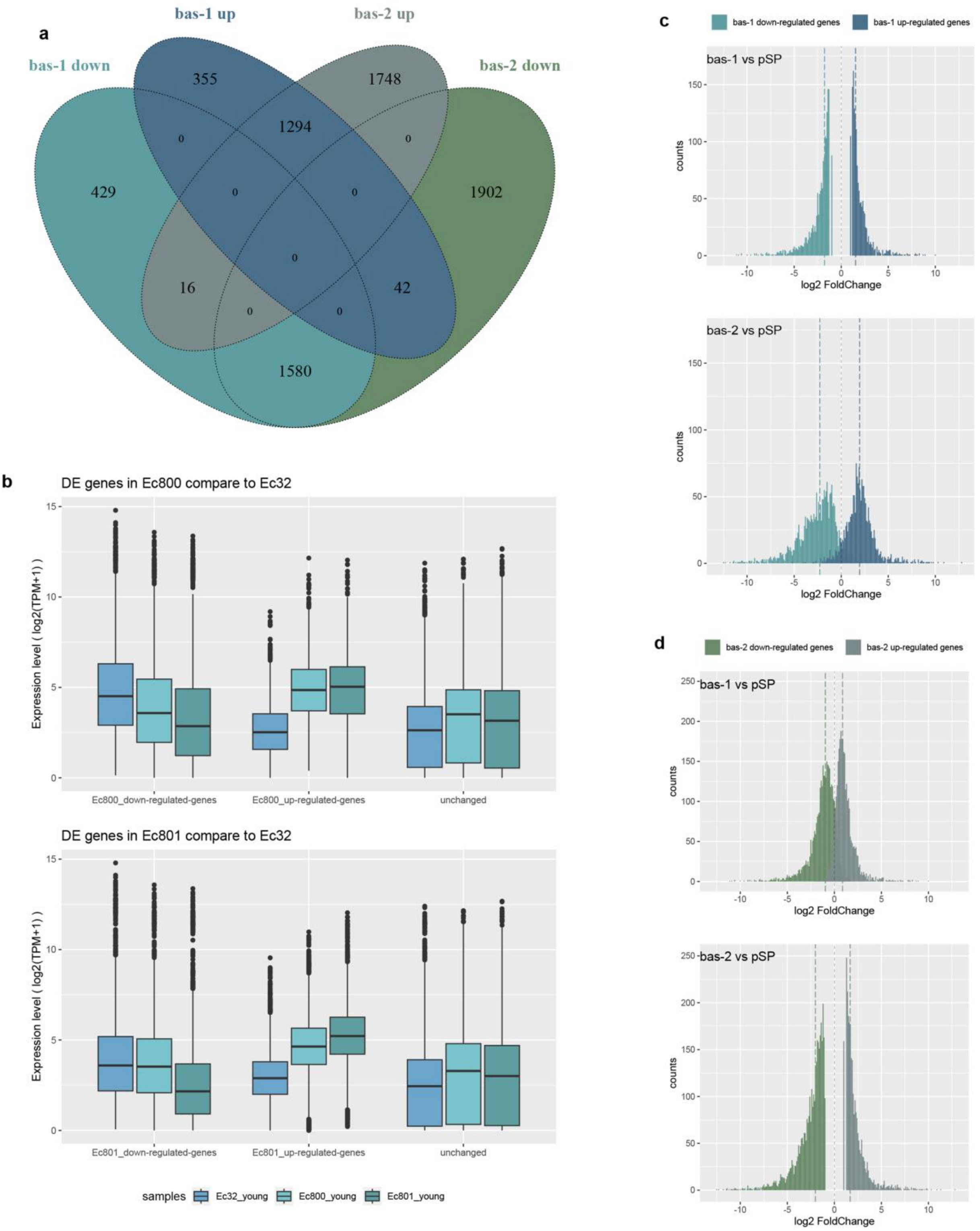

E) equivalent to Fig S7. GO term enrichment observed in DE gene sets in bas mutants compared to WT. Dot plot representation is divided according to GO term ontology classes 'Cellular Component' (**a**), ‘Molecular Function’ (**b**) and ‘Biological Processes’ (**c**).

© 2023. Published by The Company of Biologists under the terms of the Creative Commons Attribution License (https://creativecommons.org/licenses/by/4.0/).

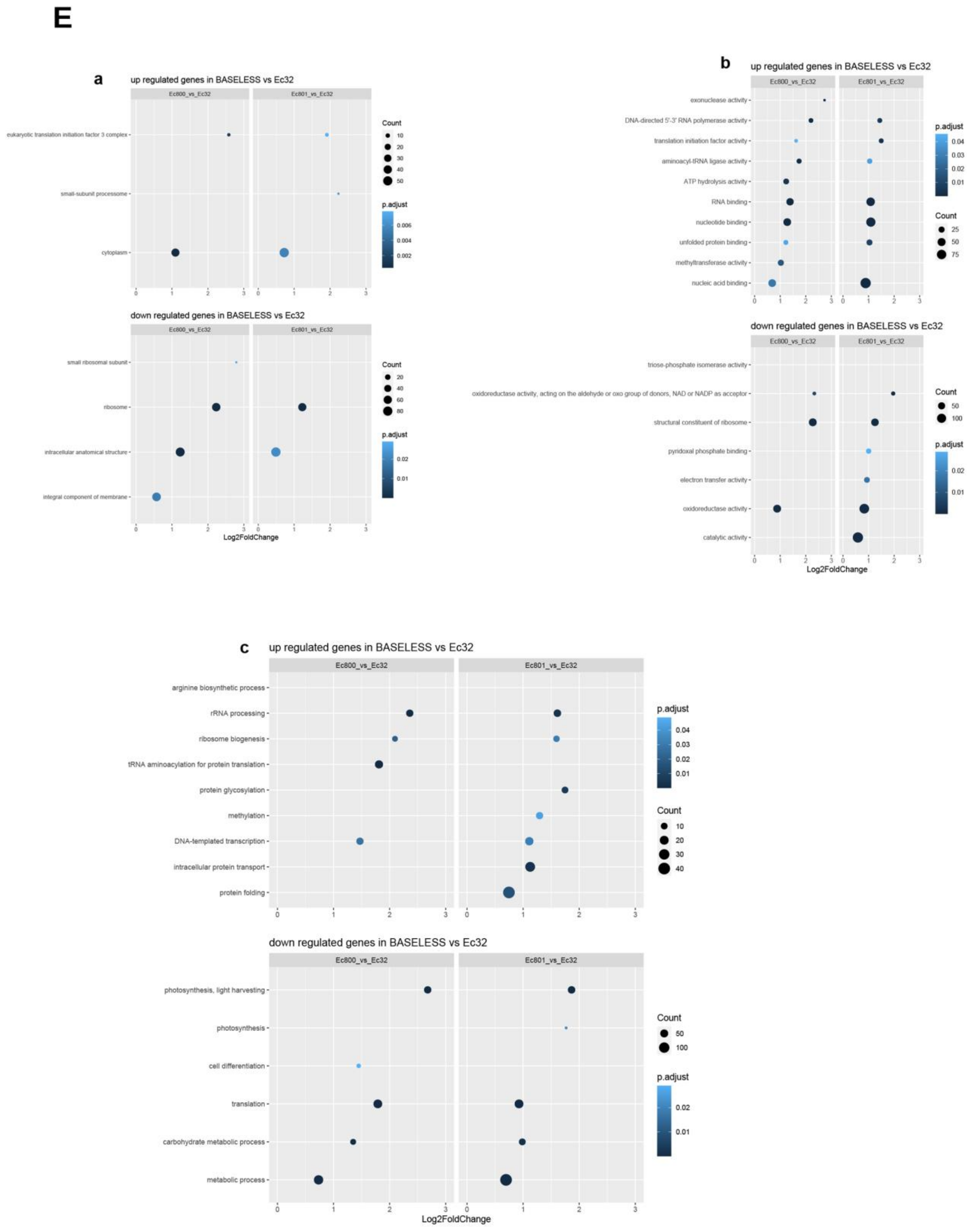

Reviewers-Figure 2. 3-replicas vs 2-replicas Differential gene expression analysis comparison

A) Differentially Expressed Genes (DEGs) lists comparison **1-2)** bas-1 (Ec800) vs WT (Ec32) **3-4)** bas-2 (Ec801) vs WT (Ec32) **1-3**) up-set showing that even genes expression change in same way in the two analysis or are specific to one analysis **2-4**) venn diagrams showing proportion of DEGs shared by the two analysis

© 2023. Published by The Company of Biologists under the terms of the Creative Commons Attribution License (https://creativecommons.org/licenses/by/4.0/).

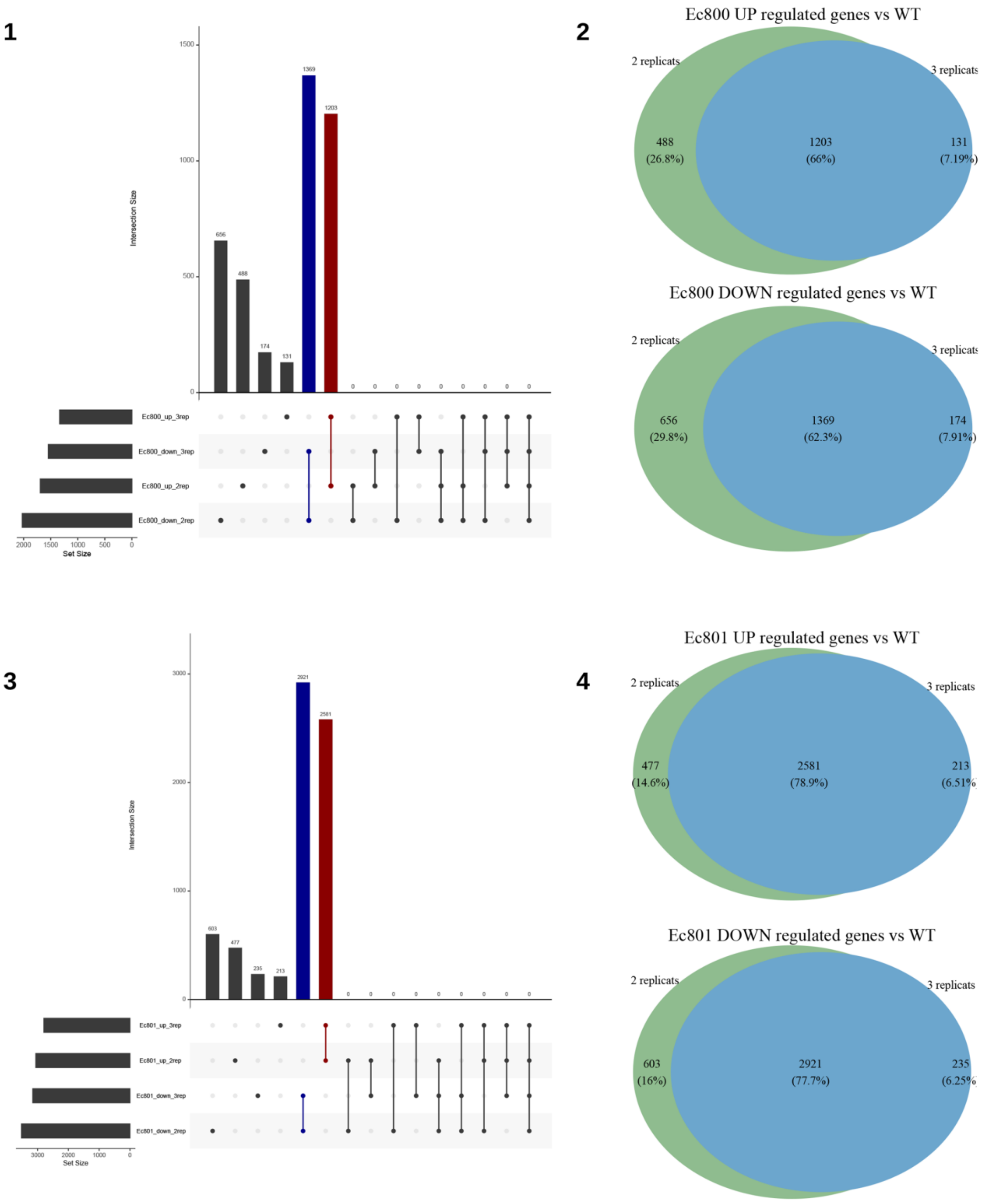

B) DEGs not shared between the two analysis are reported on vulcano plot of the 3-replicas analysis

Red cross indicate genes that are significantly DE when considering only the 2-replicas analysis but not DE in the 3-replicats analysis

Orange cross indicate genes not DE when considering only the 2-replicas analysis but significantly DE in the 3-replicas analysis

© 2023. Published by The Company of Biologists under the terms of the Creative Commons Attribution License (https://creativecommons.org/licenses/by/4.0/).

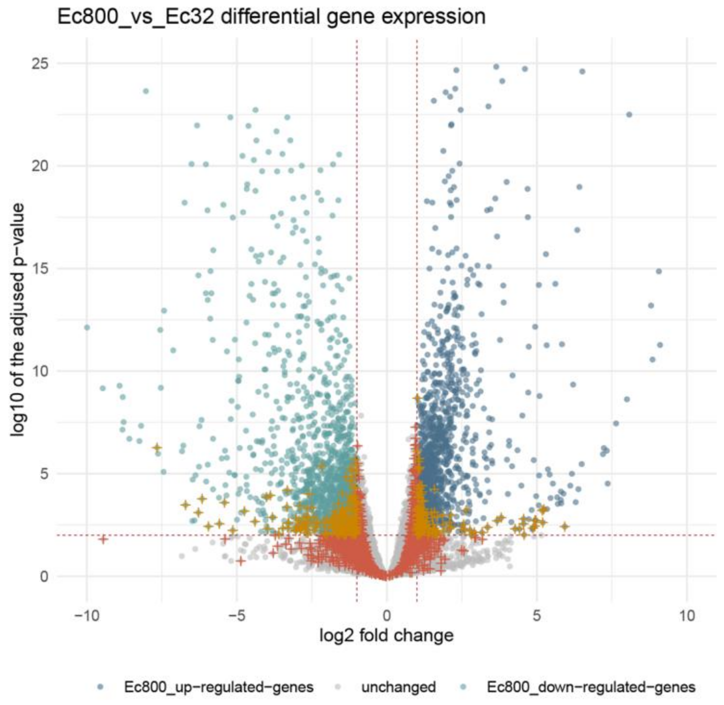

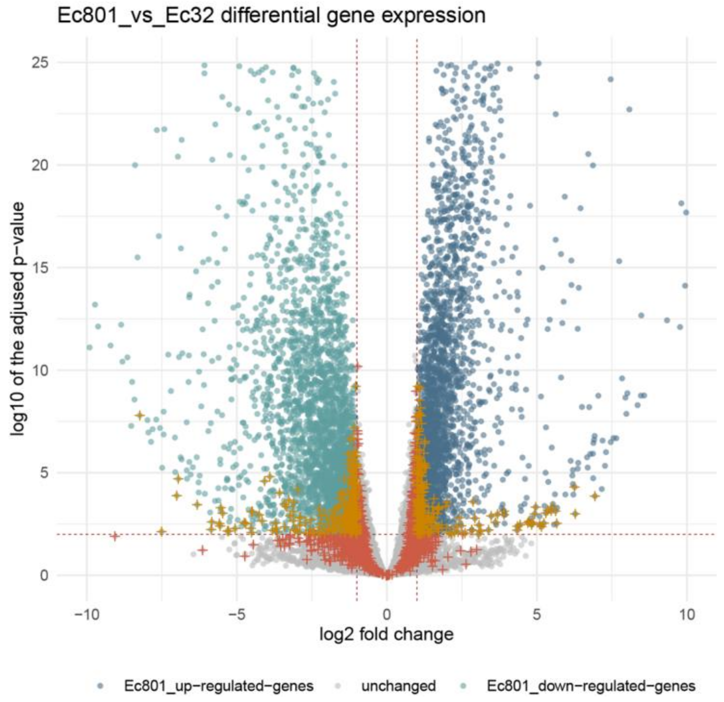

NOTE: We have removed unpublished data that had been provided for the referees in confidence.

© 2023. Published by The Company of Biologists under the terms of the Creative Commons Attribution License (https://creativecommons.org/licenses/by/4.0/).

Second decision letter

MS ID#: DEVELOP/2022/201283

MS TITLE: The baseless mutant links protein phosphatase 2A with basal cell identity in the brown alga Ectocarpus

AUTHORS: Olivier Godfroy, Min Zheng, Haiqin Yao, Agnes Henschen, Akira F Peters, Delphine Scornet, Sebastien Colin, Paolo Ronchi, Katharina Hipp, Chikako Nagasato, Taizo Motomura, J. Mark Cock, and Susana M. Coelho
ARTICLE TYPE: Research Article

I am happy to tell you that your manuscript has been accepted for publication in Development, pending our standard ethics checks. You will see that Reviewer 2 has a few suggestions for you to consider - it is entirely up to you if you wish to change any aspect of your MS to address these suggestions before submission of final files. This is not a prerequisite of publication and is entirely up to your discretion.

Reviewer 1

Advance summary and potential significance to field

The author has address my comments constructively and clearly.

Comments for the author

None

Reviewer 2

Advance summary and potential significance to field

na (covered in original review)

Comments for the author

I appreciate the authors' efforts to clarify the points raised in my previous review. I urge them to consider the following before publication.

1. The analysis of mutations in the bas-2 strain in the revision was framed as a "test" for the possibility of its phenotype being due to off target mutations. The negative results from this test were not conclusive or convincing, as they are predicated on additional assumptions about what type of mutations (coding vs non-coding regions, conserved versus non-conserved residues) have an impact, which genes are involved in meiotic development, and that all mutations were detected by resequencing. I think the analysis was worthwhile, but the only outcomes that would have refuted the authors hypothesis are a very narrow set of outcomes involving mutations in known meiotic genes. This is hardly a strong test. I would prefer language that does not invoke estimated likelihoods and instead is more neutral such as "Our data are consistent with the hypothesis that the phenotypic severity of bas-2 strains versus bas-1 strains is due to the relative severity of the bas mutations caused by each allele, but cannot rule out mutations at other loci contributing to the bas-2 phenotype."

2. The authors potential explanation for decreased photosynthetic gene expression in the bas mutants is helpful, though they should make clear whether increased pigmentation in some of the basal cells is indicative of more photosynthetic capacity (as opposed to having more photoprotective pigments). I am also surprised that so little is known about photosynthetic capacity of different Ectocarpus tissue types. Did the authors consider using their wild type apical versus

© 2023. Published by The Company of Biologists under the terms of the Creative Commons Attribution License (https://creativecommons.org/licenses/by/4.0/).

wild type basal tissue RNAseq data from the DISTAG paper to support the idea of more photosynthetic gene expression in basal tissues?

3. The meiotic defect in the heterozygous double mutant bas/BAS DIS/dis is unexpected and deserves a bit more comment or explanation. It could support a connection between the two gene products in a highly dosage sensitive process related to meiosis. I also wonder if the authors can speculate about whether meiotic development might in some way be connected to early polarity establishment during germination. In other words are the two phenotypes due to pleiotropy, or might there be an underlying commonality (e.g. microtubule defects) that connect them.

Reviewer 3

Advance summary and potential significance to field

The fact that this is a new system makes the findings more interesting and the careful characterization of mutants like these is important. This manuscript should be of interest to anyone working on the early stages of embryonic polarity and isolation/characterization of the bas mutant will be an exciting new addition to the field.

Comments for the author

I have reviewed the revised manuscript as well as the authors' attempts to address my and the other reviewers suggestions. I find the expanded explanations in the text as well as additional supplementary figures helpful in clarifying many of the points that were initially unclear. Given that this is a relatively new system there are technical limitations that can not currently be overcome. However, my concerns have been addressed.

© 2023. Published by The Company of Biologists under the terms of the Creative Commons Attribution License (https://creativecommons.org/licenses/by/4.0/).
